# Inhibiting WEE1 Selectively Kills Histone H3K36me3-Deficient Cancers by dNTP Starvation

**DOI:** 10.1016/j.ccell.2015.09.015

**Published:** 2015-11-09

**Authors:** Sophia X. Pfister, Enni Markkanen, Yanyan Jiang, Sovan Sarkar, Mick Woodcock, Giulia Orlando, Ioanna Mavrommati, Chen-Chun Pai, Lykourgos-Panagiotis Zalmas, Neele Drobnitzky, Grigory L. Dianov, Clare Verrill, Valentine M. Macaulay, Songmin Ying, Nicholas B. La Thangue, Vincenzo D’Angiolella, Anderson J. Ryan, Timothy C. Humphrey

**Affiliations:** 1 CRUK MRC Oxford Institute for Radiation Oncology, Department of Oncology, University of Oxford, Oxford OX3 7DQ, UK; 2 Institute of Pharmacology and Toxicology, Vetsuisse Faculty, Winterthurerstrasse 260, 8057 Zürich, Switzerland; 3 Department of Oncology, University of Oxford, Old Road Campus Research Building, Oxford OX3 7DQ, UK; 4 Institute of Cytology and Genetics RAS, Novosibirsk 630090, Russia; 5 Department of Cellular Pathology, Oxford University Hospitals NHS Trust, John Radcliffe Hospital, Oxford OX3 9DU, UK; 6 Oxford Cancer and Haematology Centre, Oxford University Hospitals NHS Trust, Churchill Hospital, Oxford OX3 7LJ, UK; 7 Department of Respiratory and Critical Care Medicine of the Second Affiliated Hospital and Department of Pharmacology, Zhejiang University School of Medicine, Hangzhou 310058, China

**Keywords:** cancer, synthetic lethality, epigenetic target, histone modification, SETD2, H3K36me3, WEE1 inhibitor, AZD1775, ATR inhibitor, CHK1 inhibitor, DNA replication, RRM2, CDK, KDM4A, H3.3K36M, iPOND, ChIP

## Abstract

Histone H3K36 trimethylation (H3K36me3) is frequently lost in multiple cancer types, identifying it as an important therapeutic target. Here we identify a synthetic lethal interaction in which H3K36me3-deficient cancers are acutely sensitive to WEE1 inhibition. We show that RRM2, a ribonucleotide reductase subunit, is the target of this synthetic lethal interaction. RRM2 is regulated by two pathways here: first, H3K36me3 facilitates *RRM2* expression through transcription initiation factor recruitment; second, WEE1 inhibition degrades RRM2 through untimely CDK activation. Therefore, WEE1 inhibition in H3K36me3-deficient cells results in RRM2 reduction, critical dNTP depletion, S-phase arrest, and apoptosis. Accordingly, this synthetic lethality is suppressed by increasing RRM2 expression or inhibiting RRM2 degradation. Finally, we demonstrate that WEE1 inhibitor AZD1775 regresses H3K36me3-deficient tumor xenografts.

## Highlights

• WEE1 inhibition selectively kills H3K36me3-deficient cancer cells
• These cells are killed through dNTP starvation because of RRM2 depletion
• RRM2 is regulated by H3K36me3 through transcription and WEE1 via degradation
• WEE1 inhibitor AZD1775 regresses H3K36me3-deficient tumors in vivo

## Graphical Abstract

**Figure:**
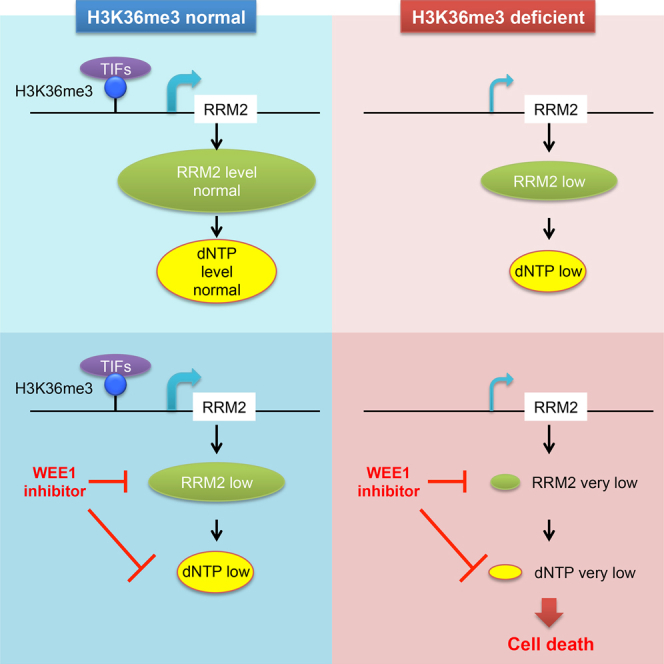

## Teaser

Pfister et al. show that WEE1 inhibition selectively kills H3K36me3-deficient cancer cells through dNTP starvation resulting from RRM2 depletion. Pfister et al. further show that H3K36me3 facilitates *RRM2* transcription whereas WEE1 inhibition promotes RRM2 degradation via CDK activation.

## Significance

**Despite the high prevalence and poor prognosis associated with H3K36me3 loss, there is no therapy targeting H3K36me3-deficient cancers. Here we show these cancers can be targeted by inhibition of WEE1. We identify distinct roles for H3K36me3 and WEE1 in RRM2 regulation, nucleotide pool maintenance, and DNA replication. Our proposed therapy is based on synthetic lethality, which provides a less toxic and more effective treatment because it specifically targets cancer cells. H3K36me3 loss can be used as a predictive biomarker, which identifies multiple genetic mutations, and enables patient selection through immunohistochemistry. Finally, because the WEE1 inhibitor, for which we describe a target, is in clinical trials, we anticipate that these findings will be of immediate clinical relevance.**

## Introduction

Trimethylation of histone H3K36 (H3K36me3) is an epigenetic mark usually associated with actively transcribed genes (Shilatifard, 2006), for which proposed functions include DNA repair (Aymard et al., 2014, Carvalho et al., 2014, Pfister et al., 2014), chromatin structure modulation during elongation (Carvalho et al., 2013), and stem cell regulation (Zhang et al., 2014, Zhu et al., 2014). Multiple mutations can cause loss of H3K36me3: loss of the tumor suppressor SETD2 (the sole methyltransferase for H3K36me3), overexpression of the oncogene KDM4A (which demethylates H3K36me3), or mutation of histone H3.3 (G34V/R or K36M) (Lewis et al., 2013). Importantly, SETD2 under-expression and mutation are associated with poor prognosis in breast cancer (Al Sarakbi et al., 2009) and renal cancer (Hakimi et al., 2013), and KDM4A overexpression is associated with poor patient outcome in ovarian cancer (Black et al., 2013). SETD2 mutations and KDM4A overexpression are together observed in more than 10% of cancers in kidney, large intestines, endometrium, and ovary (Berry and Janknecht, 2013, Dalgliesh et al., 2010, Gerlinger et al., 2012). Notably, in pediatric high-grade gliomas, H3K36me3 is lost in 54% of cases (Fontebasso et al., 2013). Despite its frequent loss and association with poor prognosis, there is no therapy targeting H3K36me3-deficient cancers.

The WEE1 kinase inhibits the activities of cyclin-dependent kinases CDK1 and CDK2 through tyrosine 15 phosphorylation (Parker and Piwnica-Worms, 1992, Watanabe et al., 1995). Inhibition of WEE1 promotes unscheduled mitotic entry through CDK1 activation, leading to loss of genome integrity (Tominaga et al., 2006). Recently, WEE1 inhibition has also been shown to induce replication stress through CDK1/2-dependent aberrant firing of replication origins and subsequent nucleotide shortage (Beck et al., 2010, Beck et al., 2012). Although studies show that WEE1 inhibition sensitizes p53-deficient cells to DNA damaging agents (Hirai et al., 2009), others argue that the chemosensitization is independent of p53 status (Van Linden et al., 2013). The WEE1 inhibitor AZD1775 (MK1775) is currently in multiple phase II clinical trials in combination with DNA-damaging agents (http://www.clinicaltrials.gov), but insights into the targeted use of this inhibitor are limited.

One way to target genetic defects in cancer is through synthetic lethality. Synthetic lethality describes a genetic interaction between two genes or two pathways, where loss of either one alone has little effect on cell viability, but the simultaneous loss of both results in cell death. Therefore, synthetic lethal interactions can be exploited to selectively kill cancer cells that carry mutations in one of the genes in the interaction by chemically inhibiting the second gene product (Chan and Giaccia, 2011). Here we describe the targeting of H3K36me3-deficient cancers by exploiting a synthetic lethal interaction with WEE1 inhibition.

## Results

### H3K36me3-Deficient Cancers Are Hypersensitive to WEE1 Inhibition

From our unpublished results, we know that the loss of Set2 (a SETD2 ortholog) is synthetically lethal with the loss of Wee1 (a WEE1 ortholog) in *Schizosaccharomyces pombe*. We therefore tested whether SETD2-deficient human cells can be selectively killed by inhibiting WEE1.

**Figure 1:**
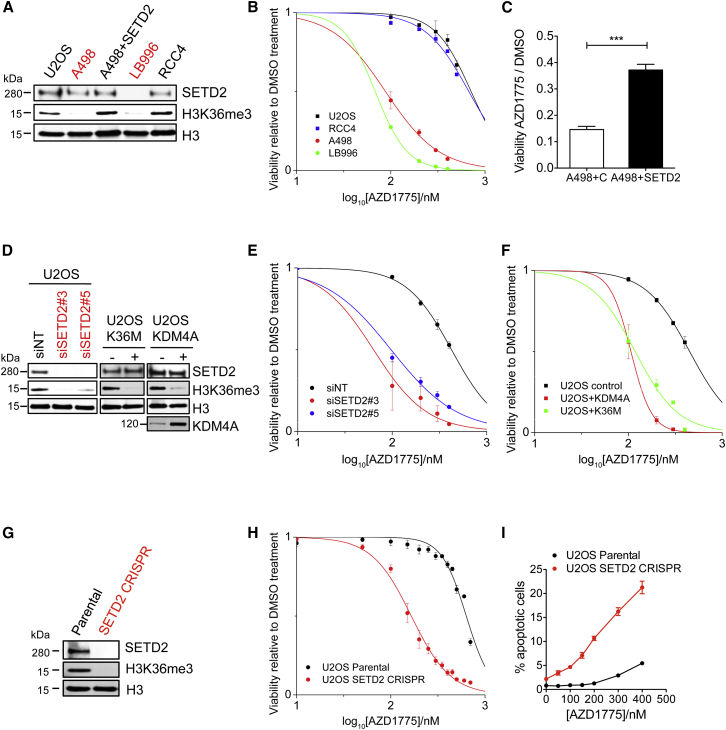
WEE1 Inhibition Selectively Kills H3K36me3-Deficient Cancer Cells (A) Western blot analysis of SETD2 and H3K36me3 levels in A498, LB996, RCC4, and U2OS cells. A498 + SETD2 are A498 cells stably expressing a *SETD2* cDNA. (B) Viability curves of SETD2 wild-type (RCC4, U2OS) and SETD2-deficient (A498, LB996) cells after exposure to WEE1 inhibitor AZD1775 (5 days). (C) Viability of A498 cells expressing either an empty vector (A498 + C) or *SETD2* cDNA (A498 + SETD2) after exposure to AZD1775 (200 nM) (72 hr). (D) Western blot analysis of SETD2, KDM4A, and H3K36me3 in U2OS cells transfected with either control siRNA (siNT) or SETD2 siRNAs (siSETD2#3, siSETD2#5), and in U2OS cells stably expressing H3.3K36M or KDM4A. (E) Viability curves of U2OS cells transfected with either control siRNA (siNT) or SETD2 siRNAs (siSETD2#3 and siSETD2#5) (48 hr) and exposed to AZD1775 (5 days). (F) Viability curves of U2OS cells expressing either an empty vector, H3.3K36M, or KDM4A after exposure to AZD1775 (5 days). (G) Western blot analysis of SETD2 and H3K36me3 levels in U2OS parental cells or U2OS cells with CRISPR knockout of SETD2. (H) Viability curves of U2OS parental cells and U2OS SETD2 CRISPR knockout cells after exposure to AZD1775 (5 days). (I) Percentage of apoptotic cells after exposure to AZD1775 (48 hr). For all graphs in Figure 1, data are presented as mean ± SEM, n = 3 independent experiments. ^∗∗∗^p < 0.001, unpaired and two-tailed t test was used. See also Figure S1.

With four different approaches, we demonstrate that H3K36me3-deficient cancer cells are hypersensitive to WEE1 inhibition. First, we found that two naturally occurring SETD2-deficient cell lines (A498 and LB996) were hypersensitive to AZD1775 (A498 half-maximal inhibitory concentration [IC_50_] = 87 nM, LB996 IC_50_ = 68 nM versus RCC4 IC_50_ = 673 nM, U2OS IC_50_ = 712 nM) (p < 0.0001) (Figures 1A, 1B, and S1A). A498, LB996, and RCC4 are renal cell carcinoma cell lines; U2OS is an osteosarcoma cell line that is suitable for genetic manipulation. A498 expresses a near full-length non-functional SETD2 protein, whereas LB996 does not express the SETD2 protein. Expressing *SETD2* cDNA in A498 cells restored H3K36me3 levels and reduced sensitivity to AZD1775 (Figures 1A and 1C). Second, SETD2 knockdown with two independent siRNAs sensitized cells to AZD1775 (Figures 1D and 1E). Third, reduction of H3K36me3 was also achieved by overexpressing the demethylase KDM4A and by expressing a mutant histone H3.3K36M (Figure 1D). In both cases, U2OS cells were sensitized to AZD1775 (KDM4A IC_50_ = 106 nM, K36M IC_50_ = 117 nM versus control IC_50_ > 400 nM) (Figure 1F). Lastly, we generated a SETD2-knockout cell line using CRISPR technology, where the gRNA-guided DNA break led to a frameshift mutation and a premature stop codon in both *SETD2* alleles, resulting in loss of the SETD2 protein (Figures 1G, S1B, and S1C). The SETD2-knockout U2OS cells were hypersensitive to AZD1775 compared to the parental SETD2 wild-type U2OS cells (CRISPR IC_50_ = 151 nM versus parental IC_50_ = 615 nM) (p < 0.0001) (Figure 1H). This effect was not only due to growth inhibition, but also cell killing, as evidenced by a 12-fold difference in clonogenic survival (CRISPR IC_50_ = 10 nM versus parental IC_50_ = 128 nM) (Figure S1D), and an up to 8-fold increase in apoptosis (Figure 1I). Moreover, siRNA knockdown of WEE1 selectively killed CRISPR SETD2-knockout cells (Figure S1E), and combining AZD1775 and WEE1 siRNA showed epistasis (Figure S1F), confirming that it is WEE1 inhibition that selectively kills H3K36me3-deficient cells. We confirmed that WEE1 is inhibited by AZD1775 by western blotting with pCDK1 Tyr15 and pan-CDK substrates (Figure S1G), and that at the doses used, AZD1775 was not inhibiting MYT1 (a kinase related to WEE1) (Figure S1H). Together, results from the four different approaches above strongly suggest a synthetic lethal interaction between H3K36me3 loss and WEE1 inhibition.

### WEE1 Inhibition Abolishes DNA Replication in SETD2-Deficient Cells

**Figure 2:**
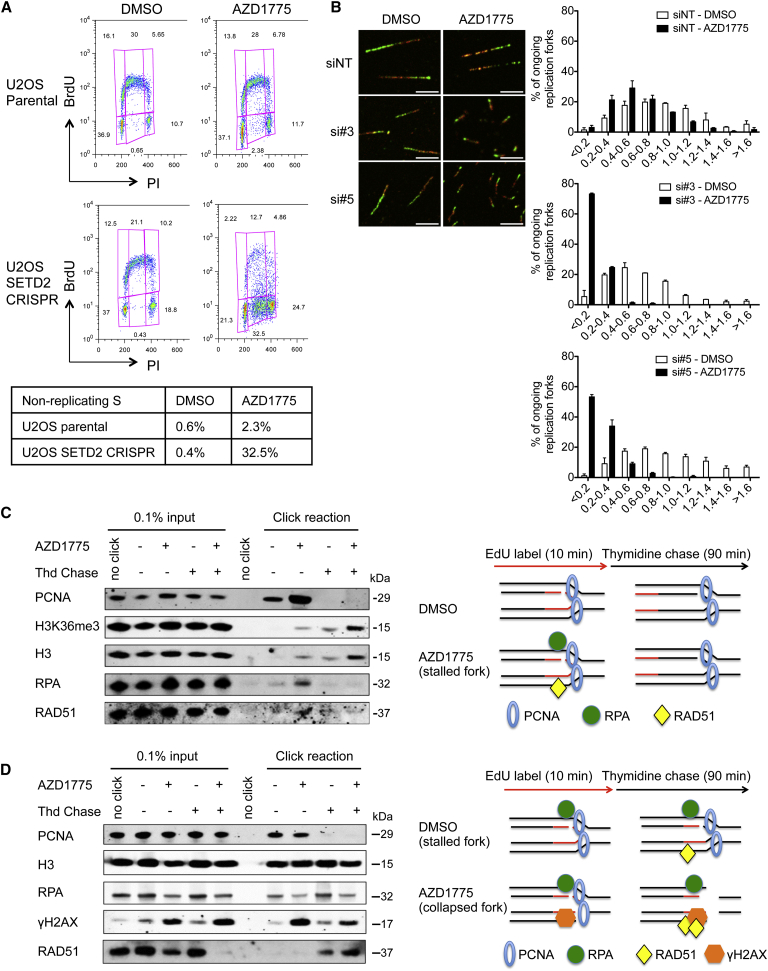
WEE1 Inhibitor AZD1775 Abolishes DNA Replication in SETD2-Deficient Cells (A) BrdU FACS analysis of the cell cycle distribution of U2OS wild-type and U2OS SETD2 CRISPR knockout cells after exposure to DMSO (0.02%) or AZD1775 (200 nM) (48 hr). (B) DNA fiber analysis of replication fork velocity in U2OS cells transfected with control siRNA (siNT) or SETD2 siRNAs (si#3 and si#5) (48 hr) prior to treatment with DMSO or AZD1775 (200 nM) (48 hr). Representative images of the replication track after treatments; scale bar represents 5 μm (left). The distribution of fork velocity was analyzed from >100 ongoing replication forks in each condition (right). Data are presented as mean ± SEM, n = 3 independent experiments. (C and D) U2OS (C) and A498 (D) cells were treated with DMSO or AZD1775 (200 nM, 24 hr), pulsed with EdU (10 min), and harvested either immediately or after thymidine chase (Thd Chase) (90 min) and analyzed by western blots (left). Graphical representations of the iPOND data are shown on the right. Click reaction was used to conjugate biotin to nascent DNA. No click acted as control with biotin azide omitted. See also Figure S2.

We next examined the mechanism underlying this selective killing of SETD2-deficient cells, and observed a significant disturbance in S-phase. In particular, WEE1 inhibitor AZD1775 forced 32% of the CRISPR SETD2-knockout cells to accumulate as non-replicating S-phase cells (exhibiting a DNA content between 2N and 4N, but not incorporating the synthetic nucleoside bromodeoxyuridine [BrdU]), whereas it had no effect on U2OS parental cells (Figure 2A). The same effect was observed in SETD2-deficient A498 cells: 40% of A498 cells accumulated in non-replicating S-phase (Figure S2A). To study the progression through S-phase, we pulse-labeled U2OS and A498 cells with BrdU and measured the cell cycle progression of the labeled cells every 2 hr. We found that while AZD1775 treatment had no effect on U2OS cells, it arrested A498’s progression through S-phase, leading to a 114-hr S-phase (calculated according to published protocol [Begg et al., 1985]) (Figure S2B). In addition, WEE1 inhibition significantly increased replication stress in SETD2-depleted U2OS cells, as shown by a 3-fold increase in pan-nuclear γH2AX staining compared to AZD1775-treated control cells (Figure S2C). Consistently, in SETD2-knockout U2OS cells, AZD1775 induced a 10-fold increase in both phospho-CHK1 and phospho-RPA staining (indicators of replication stress) compared to U2OS parental cells (Figure S2D). These data suggest that the synthetic lethality resulted from inhibition of DNA replication.

To understand the cause of S-phase arrest, we depicted the progression of individual replication forks using the DNA fiber assay. In U2OS cells, fork velocity was mildly reduced upon either SETD2 depletion or AZD1775 treatment (from an average of 0.6–0.8 kb/min to 0.4–0.6 kb/min in both cases) (Figure 2B), suggesting that both SETD2 and WEE1 are required for efficient DNA replication. Strikingly, combining SETD2 depletion with AZD1775 treatment abolished fork progression (average fork velocity < 0.2 kb/min) (Figure 2B) and significantly increased fork stalling, as demonstrated by a 3-fold increase in the percentage of stalled forks compared to AZD1775 treatment alone (measured by fiber tracks that only contained the first label) (Figure S2E).

To study the molecular events at stalled replication forks, we used iPOND (isolation of proteins on nascent DNA) (Sirbu et al., 2012). In control cells (SETD2-proficient U2OS), AZD1775 treatment resulted in transient RPA recruitment to replication forks, which disappeared after 90 min following thymidine chase (Figure 2C). No γH2AX was detected, suggesting that AZD1775-induced replication stress was efficiently resolved. In contrast, SETD2-deficient cells (A498) showed RPA accumulation at forks even without AZD1775 treatment, indicating that SETD2 loss generated replication stress. Furthermore, upon AZD1775 treatment, γH2AX, RAD51 and RPA were recruited to the replication forks, and remained after 90 min (Figure 2D). These experiments suggest that SETD2 depletion leads to fork stalling, and upon additional WEE1 inhibition, stalled forks collapse. Further investigation suggested that one of the mediators of the fork collapse is the endonuclease MUS81. MUS81 is known to cleave stalled replication forks, resulting in DNA double-stranded breaks (Hanada et al., 2007), which become substrates for fork restart (Shimura et al., 2008) (Figure S2F). Indeed, MUS81 shRNA knockdown significantly reduced DNA damage arising from combined depletion of SETD2 and WEE1 in U2OS cells as measured by the comet assay (Figures S2G–S2I).

### The Synthetic Lethal Interaction Is Not Due to p53 Loss or HR Deficiency

We next tested the possibility that p53 loss or homologous recombination (HR) deficiency were the cause of synthetic lethality with WEE1 inhibition. Previous publications showed that p53-deficient cells are killed by WEE1 inhibitors through mitotic catastrophe (Aarts et al., 2012). However, in our study, p53-deficiency was not the cause of the SETD2-WEE1 synthetic lethality. SETD2-deficient A498 and LB996 cells (which are sensitive to WEE1) both have wild-type p53 (Warburton et al., 2005). Moreover, SETD2 depletion did not affect p53 activity (Figure S2J). Therefore, WEE1 inhibition targets SETD2-deficient cells via a mechanism that is distinct from that of p53-deficient cells: SETD2-deficient cells exhibited S-phase arrest (Figures 2A, S2A, and S2B), but not premature mitosis (Figures S2K and S2L), whereas p53-deficient cells exhibited premature mitosis (Figures S2K and S2L), but not S-phase arrest (Figures S2M and S2N).

We and others have shown that SETD2-dependent H3K36me3 has a role in homologous recombination (HR) repair of DNA double-stranded breaks through facilitating LEDGF and CtIP binding to the chromatin (Aymard et al., 2014, Carvalho et al., 2014, Kanu et al., 2015, Pfister et al., 2014). However, HR deficiency is very unlikely to be the cause of sensitivity to WEE1 because BRCA2-deficient cells were not hypersensitive to WEE1 inhibition (Figure S2O). This is different from the recent finding that BRCA2 knockdown sensitizes p53-negative cells to WEE1 inhibition (Aarts et al., 2015): our cells are p53 positive, and yet a much greater sensitization with SETD2 depletion was observed. Second, if the role of SETD2 in HR was contributing to the synthetic lethal interaction with WEE1 inhibition, then depletion of the HR factors that are directly regulated by SETD2 (e.g., LEDGF and CtIP) should also result in synthetic lethality with WEE1 inhibition. However, when we depleted LEDGF or CtIP by siRNA, U2OS cells were not hypersensitive to the WEE1 inhibitor AZD1775, while the SETD2 siRNA control was hypersensitive (Figure S2P). Third, if the role of SETD2 in HR was contributing to this synthetic lethality, then SETD2 depletion in HR-defective cells should have no impact on AZD1775 sensitivity (i.e., SETD2 and other HR factors should be epistatic with regard to AZD1775 sensitivity). However, SETD2 depletion further sensitized BRCA2-depleted cells to AZD1775 (Figures S2Q and S2R), suggesting that SETD2 affects a different pathway from HR in this synthetic lethal interaction. Fourth, in *S. pombe*, the SETD2 ortholog Set2 is not required for HR (Pai et al., 2014) yet it is synthetic lethal with loss of Wee1. Lastly, HR-deficient cells are empirically known to arrest in G2 phase upon DNA damage (Farmer et al., 2005), but SETD2-knockout cells arrested in S-phase upon WEE1 inhibition (Figure 2A). These five pieces of evidence argue that SETD2′s role in HR is not the main contributor to its synthetic lethal interaction with WEE1 inhibition.

### H3K36me3 Facilitates *RRM2* Transcription

**Figure 3:**
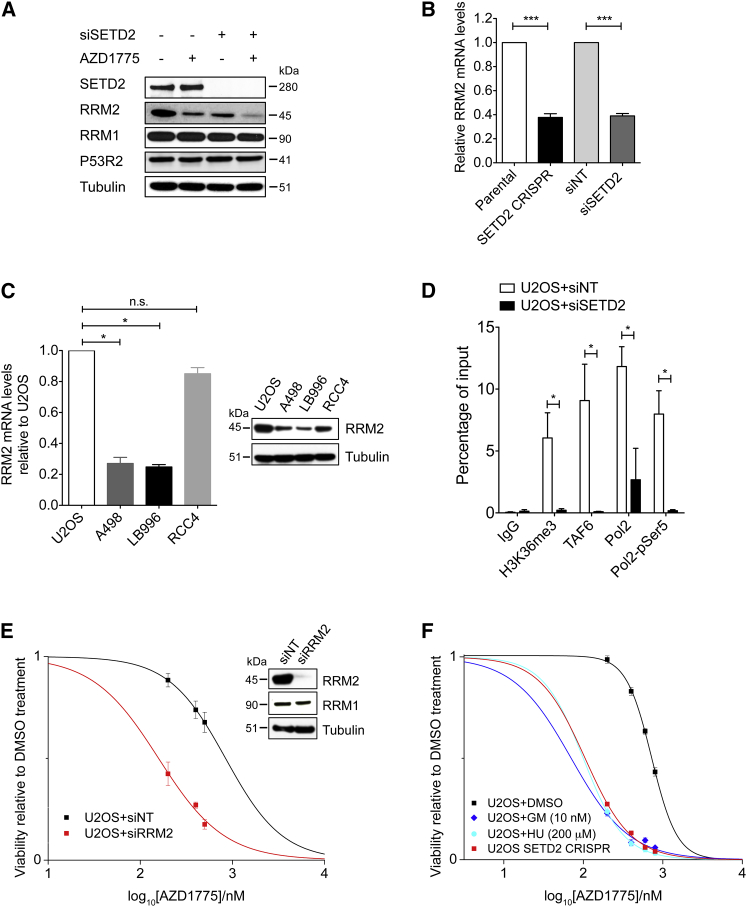
H3K36me3 Regulates *RRM2* Expression (A) Western blot analysis of RRM2, RRM1, and P53R2 protein levels in U2OS cells transfected with control (siNT) or SETD2 siRNA (siSETD2) (48 hr) and exposed to either DMSO or AZD1775 (200 nM) (24 hr). (B) qRT-PCR analysis of *RRM2* mRNA levels in siSETD2 U2OS cells (normalized to siNT) and in CRISPR SETD2-knockout U2OS cells (normalized to parental cells). (C) qRT-PCR analysis of *RRM2* mRNA levels in SETD2-deficient (A498, LB996) and proficient cells (U2OS, RCC4) (left). Western blot analysis of RRM2 protein levels in these cells (right). (D) ChIP analysis of the enrichment of H3K36me3, TAF6, RNA-Pol2, and phospho-Pol2 (Ser5) at the *RRM2* promoter in U2OS cells transfected with control siRNA (siNT) or SETD2 siRNA (siSETD2). The qPCR data are presented as percentage of input. (E) Viability curves of U2OS cells transfected with either control siRNA (siNT) or RRM2 siRNA (siRRM2) (48 hr) and exposed to AZD1775 (5 days) (left). Western blot analysis of RRM2 and RRM1 protein levels (right). (F) Viability curves of U2OS cells treated with DMSO, HU, or GM combined with indicated concentrations of AZD1775 (5 days). U2OS CRISPR SETD2-knockout cells were used as a control. For all graphs in Figure 3, data are presented as mean ± SEM, n = 3 independent experiments. ^∗∗∗^p < 0.001, ^∗∗^p < 0.01; ^∗^p < 0.05; n.s., not significant. Unpaired and two-tailed t tests were used for (D), and column statistics (one sample t test) were used for (B) and (C). See also Figure S3.

We next investigated the cause of fork stalling and S-phase arrest. It is known that loss of ribonucleotide reductase (RNR) leads to fork stalling and S-phase arrest through deoxyribonucleoside triphosphate (dNTP) depletion (Paulsen et al., 2009). Therefore, we examined the levels of RNR subunits: RRM1, RRM2, and P53R2. We found that RRM2 protein levels were reduced by both AZD1775 treatment and SETD2 knockdown (siSETD2) in U2OS, and combining them (siSETD2 + AZD1775) further depleted RRM2, whereas RRM1 and P53R2 were unaffected (Figure 3A). The same effect on RRM2 was also observed in the isogenic wild-type and CRISPR SETD2-knockout U2OS cells (Figure S3A). This reduction in RRM2 was not simply a cell cycle effect, because combining SETD2 depletion and WEE1 inhibition arrests cells in S-phase in which RRM2 levels are known to be the highest (Chabes and Thelander, 2000). In addition, SETD2 depletion does not alter the cell cycle distribution (Figure S3B) (Kanu et al., 2015, Pfister et al., 2014). This reduction was also unlikely to be due to DNA damage because RRM2 levels increase following DNA damage (D’Angiolella et al., 2012).

Synthetic lethality results from disruption of two independent pathways, which together perform an essential function (in this case DNA replication through RRM2 activity). To understand how SETD2 depletion reduces RRM2 protein levels (the first pathway), we performed qRT-PCR, and found that *RRM2* mRNA levels were reduced by 60% by either SETD2 knockdown or CRISPR SETD2-knockout (Figure 3B), suggesting that SETD2 affects the transcription of *RRM2*. This result was further confirmed in naturally occurring SETD2-deficient cell lines (A498 and LB996), where the *RRM2* transcript and protein levels were 60% lower than that in SETD2 wild-type cells (Figure 3C). Reducing H3K36me3 independently of SETD2 mutation (e.g., expressing H3.3K36M) also reduced RRM2 levels (Figure S3C).

We next explored how SETD2 affects the transcription of RRM2. Two genome-wide studies suggested a link between H3K36me3 and RRM2 promoter activity: First, a chromatin immunoprecipitation (ChIP) mass spectrometry study found that 9 of 16 TBP-associated factors (TAFs) required for the initiation of transcription by RNA polymerase II (Pol2) are enriched at the histone mark H3K36me3 (Vermeulen et al., 2010). Second, analysis of a ChIP-seq database (ENCODE) from multiple cancer cell lines showed enrichment for H3K36me3 at the *RRM2* promoter (Figure S3D). Consistent with the genome-wide studies, we found, using ChIP-qPCR, that H3K36me3 was present at the *RRM2* promoter (Figures 3D and S3E), and recruited TAFs to facilitate transcription initiation. Upon either SETD2 knockdown or CRISPR SETD2 knockout, we observed significantly reduced enrichment of H3K36me3, TAF6, Pol2, and pSer5-Pol2 (serine 5 phosphorylation is associated with transcription initiation) to the *RRM2* promoter (Figures 3D and S3E). The reduction in the promoter-bound Pol2 was also reflected in reduced occupancy of Pol2 throughout the gene body of *RRM2* in CRISPR SETD2-knockout cells (Figures S3F–S3H). Consistent with this, pSer5-Pol2 (associated with 5′ of the gene) and pSer2-Pol2 (associated with 3′ of the gene) were also reduced at their corresponding positions in CRISPR SETD2 knockout cells (Figures S3I and S3J). The reduction in *RRM2* transcription is not simply a global effect resulting from H3K36me3 loss: previous RNA-seq studies found only a small number of differentially expressed genes in SETD2-deficient cells (Kanu et al., 2015). Together, these data suggest that H3K36me3 modification regulates *RRM2* transcription.

If the reduction in RRM2 (as a result of H3K36me3 loss) was the cause of the sensitivity toward the WEE1 inhibitor AZD1775, then RRM2 depletion or inhibition should resemble H3K36me3 loss, and also exhibit synthetic lethality with AZD1775. Indeed, depleting RRM2 by siRNA significantly sensitized SETD2 wild-type U2OS cells toward AZD1775 (siRRM2 IC_50_ = 157 nM versus siNT IC_50_ = 842 nM) (Figure 3E). Likewise, inhibiting RRM2 by hydroxyurea (HU) or gemcitabine (GM) sensitized the U2OS cells to AZD1775 (Figure 3F). Moreover, rescue experiments show that transient expression of a *RRM2* cDNA reduced the sensitivity of SETD2-knockout cells toward AZD1775 to a similar degree as a *SETD2* cDNA (Figures S3K and S3L). The data in this section together suggest that H3K36me3 loss reduces *RRM2* transcription, which sensitizes cells to the WEE1 inhibitor AZD1775.

### WEE1 Inhibition Promotes RRM2 Degradation through CDK Activation

Next, we investigated how WEE1 inhibition reduces RRM2 protein levels (the second pathway). WEE1 restrains the activity of CDK1/2 during S-phase (Beck et al., 2012), and CDK/SCF^Cyclin F^ is known to promote the ubiquitin-mediated proteolysis of RRM2 (D’Angiolella et al., 2012). Following CDK-mediated phosphorylation of RRM2 at Thr33, Cyclin F binds RRM2 at its CY motif (residues 49–51 of RRM2), promoting RRM2 ubiquitylation and degradation via SCF^Cyclin F^. We found that WEE1 inhibition leaves CDK1/2 activity unchecked, leading to untimely RRM2 degradation when dNTP is still needed.

**Figure 4:**
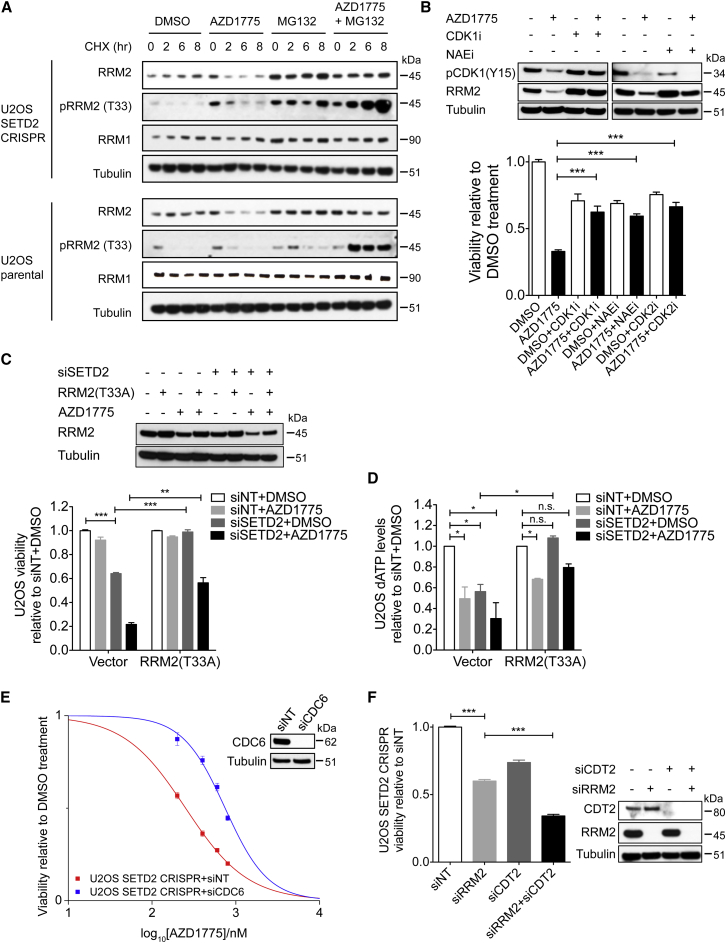
WEE1 Inhibition Promotes RRM2 Degradation and Aberrant Origin Firing (A) U2OS and U2OS CRISPR SETD2-knockout cells were treated with cycloheximide (50 μg/ml) in the presence of DMSO, AZD1775 (400 nM), MG132 (10 μM) or AZD1775 + MG132. Cells were collected at the indicated times, lysed, and immunoblotted as indicated. (B) A498 cells were treated with AZD1775 (200 nM) combined with either DMSO, neddylation inhibitor MLN4924 (NAEi) (0.1 mM), CDK1 inhibitor RO3306 (CDK1i) (10 μM) or CDK2 inhibitor (CVT-313) (3 μM). Western blot analysis of phospho-CDK1 (Y15) and RRM2 levels after indicated treatment (24 hr) (top). Viability of A498 cells after indicated treatment (72 hr) (bottom). (C) U2OS cells expressing either an empty vector (Vector) or a degradation-resistant mutant RRM2 (T33A) were transfected with control siRNA (siNT) or SETD2 siRNA (siSETD2) (48 hr) prior to treatment with either DMSO or AZD1775 (200 nM). Western blot analysis of RRM2 levels after indicated treatment (24 hr) (top), and viability of these cells after indicated treatment (96 hr) (bottom). (D) dATP levels in U2OS cells stably expressing either an empty vector (Vector) or a degradation-resistant mutant RRM2 (T33A), transfected with control siRNA (siNT) or SETD2 siRNA (siSETD2) (48 hr) prior to treatment with either DMSO or AZD1775 (200 nM) (24 hr). Data are normalized to the control (siNT + DMSO) of each cell line. (E) Viability curves of U2OS CRISPR SETD2-knockout cells transfected with either control siRNA (siNT) or CDC6 siRNA (siCDC6) (48 hr) and exposed to AZD1775 (5 days) (left). Western blot analysis of CDC6 protein levels (right). (F) Viability of U2OS CRISPR SETD2-knockout cells transfected with control siRNA (siNT), RRM2 siRNA (siRRM2), CDT2 siRNA (siCDT2), or both (siRRM2 + siCDT2) (48 hr) (left). Western blot analysis of RRM2 and CDT2 protein levels (right). For all graphs in Figure 4, data are presented as mean ± SEM, n = 3 independent experiments. ^∗∗∗^p < 0.001; ^∗∗^p < 0.01; ^∗^p < 0.05; n.s., not significant. Unpaired and two-tailed t tests were used for (B), (C), and (F), and column statistics (one sample t test) were used for (D). See also Figure S4.

First, we investigated the impact of WEE1 inhibition on RRM2 stability. Cycloheximide was used to block protein translation to allow measurement of RRM2 half-life in the presence or absence of the WEE1 inhibitor AZD1775. We found that AZD1775 treatment reduced RRM2 half-life from over 8 hr to less than 2 hr in both wild-type and CRISPR SETD2-knockout U2OS cells (Figure 4A). The reduction in half-life was accompanied by an increase in RRM2 phosphorylation at Thr33 (Figure 4A), which was shown to be mediated by CDK activity and signals it for ubiquitination and proteolysis (D’Angiolella et al., 2012). This AZD1775-induced increase in pRRM2 (T33) and reduction in RRM2 levels can also be seen without cycloheximide (Figure S4A). As a control, RRM1, whose stability is not regulated by CDKs, showed no change in half-life upon AZD1775 treatment (Figure 4A). To confirm that RRM2 half-life was reduced due to degradation, we used MG132 to block proteolysis, and observed that RRM2 half-life was no longer reduced by AZD1775, despite the increase in phosphorylation at T33 (Figure 4A). These data together suggest that WEE1 inhibition promotes RRM2 degradation by increasing its phosphorylation at T33. The impact of AZD1775 on RRM2 stability was the same for both SETD2 wild-type and knockout cells (Figure 4A), the only difference being that SETD2-knockout cells had less RRM2 to start with due to reduced transcription, which lead to critically low RRM2 levels in SETD2-knockout cells after AZD1775 treatment (Figures 3A–3D).

Next, we tested whether CDK activation (due to WEE1 inhibition) degrades RRM2 and kills SETD2-deficient cells. We found that in the presence of AZD1775, inhibiting CDK1 by RO3306 or inhibiting the NEDD8 activating enzyme with neddylation inhibitor MLN4924 (NAEi) (Soucy et al., 2009) (which blocks cullin-mediated degradation of RRM2) both restored RRM2 protein levels and viability of A498 or SETD2-knockout U2OS cells (Figures 4B and S4B). In addition to restoring cell viability, RO3306 and MLN4924 also alleviated AZD1775-induced non-replicating S-phase, replication stress, and apoptosis in SETD2-deficient cells (Figures S4C–S4E). CDK2 inhibition by CVT-313 (Brooks et al., 1997) also rescued the viability of A498 and SETD2-knockout cells (Figures 4B and S4B) and partially restored RRM2 levels (Figure S4F).

To further support our hypothesis that WEE1 regulates RRM2 degradation in S-phase by inhibiting CDK, we reasoned that inhibiting other negative regulators of CDK such as CHK1 and ATR, which also function in S-phase (Sørensen and Syljuåsen, 2012), may have a similar impact on RRM2. Indeed, inhibition of CHK1 and ATR reduced the inhibitory Y15 phosphorylation on CDK1, leading to a significant reduction in RRM2 in SETD2-deficient or depleted cells (Figure S4G). Consistent with the reduction in RRM2, inhibition of CHK1 and ATR selectively killed H3K36me3-depleted cells (including CRISPR SETD2-knockout, H3.3K36M expressing or KDM4A overexpressing cells) (Figures S4H–S4K). As with WEE1 inhibition, treatment of SETD2-deficient or depleted cells with CHK1 and ATR inhibitors resulted in non-replicating S-phase (Figure S4L) and dNTP depletion (Figure S4M). In addition, the mechanism of rescue was the same: CDK inhibition reduced the toxicity of CHK1 and ATR inhibitors toward SETD2-deficient cells (Figure S4N). These data support our hypothesis that WEE1 inhibition selectively kills H3K36me3-deficient cells by promoting CDK-dependent RRM2 degradation.

### Exogenous RRM2 Expression Rescues the Synthetic Lethality

So far, we have shown that RRM2 levels are regulated by two pathways in this context: H3K36me3 and CDK. In accordance with RRM2 being the target of this synthetic lethal interaction, we found that expressing exogenous RRM2 rescued the synthetic lethality. We expressed a mutant RRM2 (T33A) that cannot be phosphorylated by CDK and degraded by SCF^Cyclin F^ (D’Angiolella et al., 2012). Exogenous T33A expression increased RRM2 proteins to a level that was slightly lower than normal levels (Figure 4C), and suppressed the sensitivity of SETD2-knockdown or SETD2-knockout cells toward AZD1775 (Figures 4C and S3K).

In addition to T33A, the toxicity of the WEE1 inhibitor was also suppressed by another RRM2 mutant: RxI/AxA, but not by wild-type RRM2 (Figure S4O). The RxI/AxA mutation locates at the CY motif of RRM2 (residue 49–51) and abolishes its interaction with Cyclin F, thus preventing RRM2 degradation by proteolysis (D’Angiolella et al., 2012). This suggests that WEE1 inhibition promotes SCF^Cyclin F^-mediated degradation of RRM2.

As expected, the reduction in RRM2 protein levels was reflected in reduced cellular dNTP levels. dATP levels were reduced by 50% following either SETD2 depletion or AZD1775, and by 70% when these treatments were combined (Figure 4D). T33A expression, which restored RRM2 protein levels, also restored dATP levels in AZD1775-treated SETD2-depleted cells (Figure 4D). The other dNTP levels were also reduced by either SETD2 depletion or AZD1775 treatment (Figure S4P), and the reduction in dNTP levels was rescued by T33A expression (Figure S4Q). Together, these data support a model in which RRM2 is the target of the synthetic lethal interaction between H3K36me3 loss and WEE1 inhibition.

### WEE1 Inhibition Promotes Aberrant Origin Firing, Enhancing the Toxicity of RRM2 Depletion

We have shown that RRM2 is transcriptionally regulated by H3K36me3 and is degraded by WEE1 inhibition. Because WEE1 inhibition also causes aberrant origin firing through CDK activation, which (like RRM2 depletion) exhausts the dNTP pool (Beck et al., 2012), we tested whether aberrant origin firing worked together with RRM2 depletion to cause synthetic lethality. Consistent with this, we found that reducing origin firing by depleting CDC6 or CDT1 (replication licensing factors) (Beck et al., 2012, Jones et al., 2013) suppressed the sensitivity of SETD2-knockout cells toward AZD1775 (Figures 4E and S4R) and reduced the non-replicating S-phase population by 50% (Figure S4S). Furthermore, depleting CDT2 (part of the ubiquitin ligase that degrades CDT1) (Jin et al., 2006) further sensitized SETD2-knockout cells to RRM2 depletion (Figure 4F).

This proposed dual impact of WEE1 inhibition (RRM2 degradation and aberrant origin firing) suggests that the traditional inhibitors of RRM2 would be less efficient in killing H3K36me3-deficient cells. Indeed, HU or GM (two inhibitors of ribonucleotide reductase) sensitized SETD2-knockout cells, but neither achieved a level of sensitivity comparable to the WEE1 inhibitor AZD1775 (2-fold or less difference in IC_50_ for HU and GM versus over 5-fold difference in IC_50_ for AZD1775 in the same assay) (Figures S4T and S4U). These data suggest that traditional inhibitors of RRM2 (HU and GM) cannot replace AZD1775 in targeting H3K36me3-deficient cells.

### WEE1 Inhibitor AZD1775 Regresses SETD2-Deficient Tumors In Vivo

**Figure 5:**
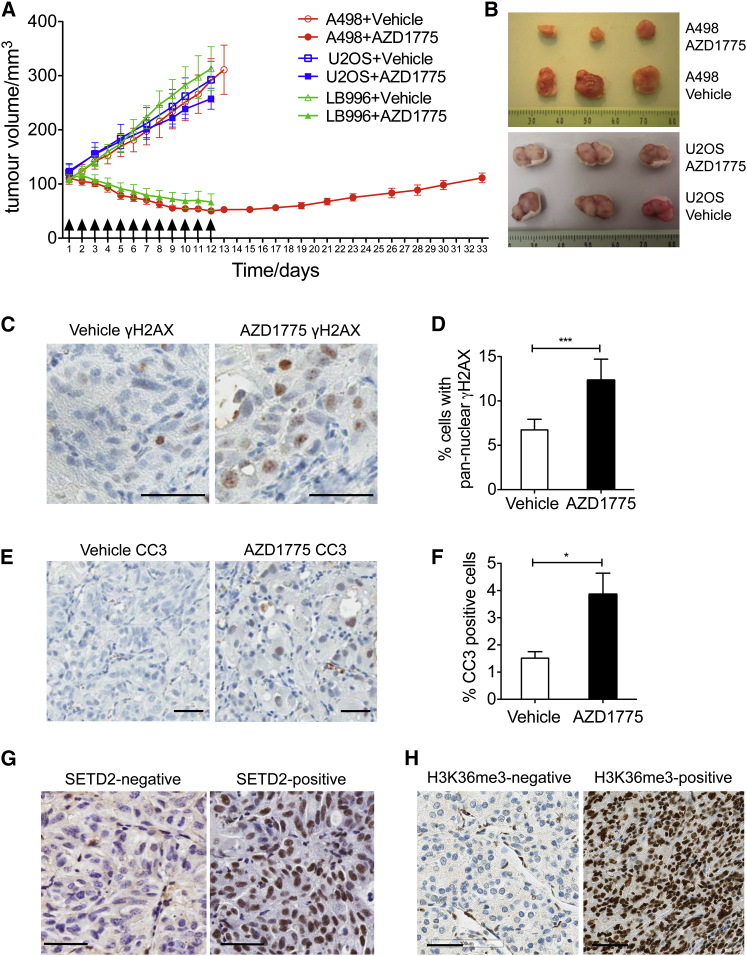
WEE1 Inhibitor AZD1775 Regresses SETD2-Deficient Tumors In Vivo (A) Tumor volumes for each treatment group, with arrows indicating the days when the inhibitors were given. Data are presented as mean ± SEM, n = 7 mice for A498 and U2OS, n = 5 mice for LB996. (B) Representative tumors in mice treated with either AZD1775 or vehicle (day 13). (C–F) Representative images of tumors generated from A498 cells (C and E) and quantification results (D and F) of immunohistochemistry analysis of pan-nuclear γH2AX (C and D) and cleaved-Caspase-3 (CC3) (E and F) levels in tumors (day 13). Scale bar represents 50 μm. Data are presented as mean ± SEM, n = 3 tumors. ^∗∗∗^p < 0.001; ^∗^p < 0.05; unpaired and two-tailed t tests were used. (G) Immunohistochemistry staining with anti-H3K36me3 antibody to distinguish SETD2-deficient from SETD2-proficient xenografts. Scale bar represents 50 μm. (H) Representative images of H3K36me3-negative and H3K36me3-positive tumors identified by immunohistochemistry staining of human renal cancer patient tissue microarray. Scale bar represents 50 μm. See also Figure S5.

The selective and potent killing of SETD2-deficient cells by AZD1775 suggested that AZD1775 could potentially be used to treat H3K36me3-deficient cancers. To test the in vivo efficacy of AZD1775, we established tumor xenografts in nude mice. Upon AZD1775 treatment, all seven tumors generated from SETD2-deficient A498 cells regressed from day 3 onward, resulting in a 5.8-fold reduction in tumor size compared to vehicle-treated control animals (tumor size at day 12 = 50.2 ± 4.7 mm^3^ versus 291.2 ± 40.0 mm^3^, p < 0.0001) (Figures 5A and 5B). Consistent with the observation from A498 cells, upon AZD1775 treatment, all five tumors generated from another SETD2-deficient cell line (LB996) regressed from day 3 onward, resulting in a 4.7-fold reduction in tumor size compared to vehicle-treated control animals (tumor size at day 12 = 66.5 ± 15.3 mm^3^ versus 313.3 ± 40.2 mm^3^, p < 0.0001) (Figure 5A). In contrast, AZD1775 treatment of tumors generated from SETD2-proficient U2OS cells did not have any significant effect on tumor growth (tumor size at day 12 = 257.5 ± 19.3 mm^3^ versus 292.5 ± 34.3 mm^3^, p = 0.37) (Figures 5A and 5B).

Consistent with the in vitro data, AZD1775-treated SETD2-deficient tumors showed significantly greater levels of replication stress (measured by γH2AX pan-nuclear staining) (Figures 5C and 5D), accompanied by increased apoptosis (measured by cleaved caspase-3) compared with vehicle-treated control (Figures 5E and 5F). No difference in body weight was observed between AZD1775-treated mice and the vehicle-treated control (Figure S5), suggesting that AZD1775 was well tolerated.

Finally, to identify patients who could benefit from this treatment, we sought a single biomarker to identify all the genetic mutations that lead to loss of H3K36me3. Using a monoclonal antibody against H3K36me3, we were able to distinguish SETD2-deficient from SETD2-proficient tumor xenografts by immunohistochemistry (IHC) (Figure 5G). Importantly, the same IHC method worked on fixed patient tissue microarrays (TMAs), thus enabling us to identify cancer patients with H3K36me3-negative tumors. Using this method, we analyzed renal cancer TMAs and found that 22 out of 122 (18%) cancers showed complete loss of H3K36me3 (while stromal tissue showed normal staining for H3K36me3) (Figure 5H). This high prevalence of H3K36me3 loss further supports the medical intervention of treating H3K36me3-deficient cancers with the WEE1 inhibitor AZD1775.

## Discussion

**Figure 6:**
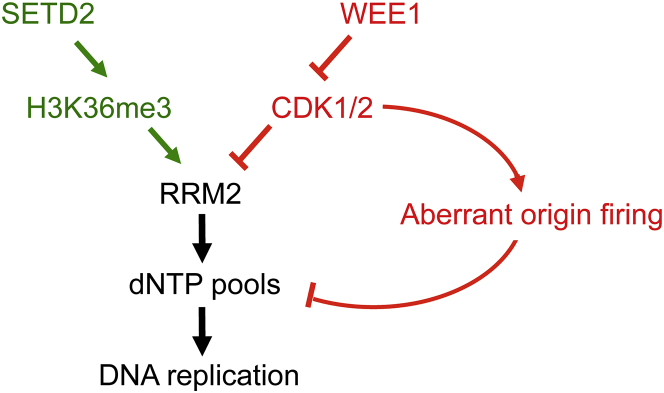
Schematic Overview of the Synthetic Lethal Interaction between H3K36me3 Loss and WEE1 Inhibition RRM2 is regulated by two pathways. In the first, SETD2 catalyzes histone H3K36me3, which promotes *RRM2* expression. In the second, WEE1 negatively regulates CDK activity and upon WEE1 inhibition, hyperactive CDK promotes RRM2 degradation and aberrant origin firing. Therefore, WEE1 inhibition in H3K36me3-deficient cells leads to critically reduced dNTP pool levels, resulting in replication stress and cell death.

Despite the frequent loss of histone H3K36me3 in multiple cancer types and its association with poor patient outcome, there is no therapy targeting H3K36me3-deficient cancers. Although WEE1 inhibitors are currently in clinical trials, insights into determinants of sensitivity to WEE1 inhibitors are limited. Here, we identified a synthetic lethal interaction between loss of H3K36me3 and inhibition of WEE1. We established RRM2, a subunit of ribonucleotide reductase, to be the target of this synthetic lethal interaction, and showed it to be regulated by two pathways in this context. Disrupting the first pathway through H3K36me3 depletion reduces RRM2 expression and dNTP production. Disrupting the second pathway through WEE1 inhibition leads to aberrant CDK activity in S-phase, resulting in untimely RRM2 degradation, aberrant origin firing, and reduced dNTP pool levels. Simultaneous disruption of both pathways leads to critically low RRM2 levels, dNTP pool depletion, inhibition of DNA replication, and cell death (Figure 6).

We have identified a role for SETD2-dependent H3K36me3 in facilitating RRM2-dependent nucleotide synthesis and efficient DNA replication. In all H3K36me3-deficient cell lines tested, RRM2 levels were consistently reduced. This is not simply a cell cycle effect because H3K36me3-depletion had no effect on the cell cycle (Aymard et al., 2014, Kanu et al., 2015, Pfister et al., 2014). Nor is this a DNA damage effect because RRM2 levels increase after DNA damage (D’Angiolella et al., 2012). Furthermore, this is not a result of global H3K36me3 loss because SETD2 depletion only changes the expression of a small subset of genes (Kanu et al., 2015).

The role of H3K36me3 in transcription initiation of RRM2 is perhaps unexpected, because H3K36me3 has previously been shown to be present mainly at the coding region of genes. However, our ChIP experiments and analysis of the ENCODE as well as ChIP mass-spectrometry data suggest a role in transcription: H3K36me3 is present at the promoter of *RRM2* and recruits transcription initiation factors (e.g., TAFs). This finding is in accordance with the recent report of a role for murine SETD2 in transcriptional initiation of genes that are involved in endothelial differentiation (Zhang et al., 2014). In the absence of H3K36me3, RRM2 and dNTP levels are reduced, resulting in low levels of replication stress, but without obvious cell cycle effects. However, we anticipate that the replication stress generated by H3K36me3 loss promotes genome instability, an enabling characteristic of cancer. This finding, in conjunction with additional recently defined roles for H3K36me3 in maintaining genome stability (Kanu et al., 2015, Pfister et al., 2014, Sato et al., 2013, Zhu et al., 2014) further suggests how loss of this histone mark can promote tumorigenesis.

Our findings also suggest that the RRM2 inhibitors HU and GM cannot replace AZD1775 in targeting H3K36me3-deficient cells. This is because a second effect of WEE1 inhibition (aberrant origin firing) works together with RRM2 degradation to kill H3K36me3-deficient cells. We showed that depleting RRM2 without increasing origin firing (e.g., by siRRM2, HU or GM), or increasing origin firing without depleting RRM2 (e.g., by siCDT2 alone), is less efficient in killing H3K36me3-deficient cells than combining aberrant origin firing with RRM2 depletion. These findings advance our current understanding of WEE1 inhibition, showing that it induces replication stress through RRM2 depletion as well as aberrant origin firing (Beck et al., 2012). The synergy between AZD1775 and HU/GM further supports our hypothesis that dNTP levels are the main mechanism underlying the synthetic lethal interaction. However, our data indicate that AZD1775 + HU/GM is likely to be a lethal combination in the clinic, being toxic for all cells. In contrast, AZD1775 treatment selectively affects cancer cells by targeting H3K36me3-deficiency.

In summary, we define a synthetic lethality between H3K36me3 loss and WEE1 inhibition, and its underlying mechanisms, by a variety of experimental approaches in many cell lines. We further demonstrate that H3K36me3-deficient cancers can be targeted in vivo by exploiting this synthetic lethal relationship. The sensitivity to the WEE1 inhibitor results through DNA replication stress because of RRM2 depletion, suggesting that other cancers exhibiting such replication stress may be targeted similarly. Because the WEE1 inhibitor, AZD1775, is already in clinical trials, and because we have identified a testable predictive biomarker for loss of H3K36me3, we expect our findings to be of immediate clinical relevance.

## Experimental Procedures

### Generation of CRISPR SETD2 Knockout Cells

The CRISPR plasmid was purchased from Horizon Discovery Group, and the plasmid (pD1301) contains the chimeric gRNA scaffold, Cas9 and DasherGFP. The target sequence of the gRNA is ACTCTGATCGTCGCTACCAT (first exon of *SETD2*). The plasmid was transfected into U2OS cells using Fugene6 (Promega) and single-cell colonies of GFP-positive cells were selected and validated by western blotting and genomic PCR.

### iPOND

A total of 3 × 10^7^ cells were treated with the AZD1775 or DMSO (24 hr) and pulsed with 10 μM EdU (10 min), after which they were either crosslinked (1% formaldehyde in PBS), or washed and released into fresh medium containing 10 μM thymidine and AZD1775/DMSO for additional 90 min before crosslinking. The click reaction was performed according to the published protocol (Sirbu et al., 2012). Samples were boiled (30 min) to de-crosslink proteins before western blotting.

### Measurement of dNTP Pools

Nucleotides were extracted by incubating cells in 60% methanol (1 hr) at −20°C. Samples were boiled (3 min) and centrifuged at 13,000 × *g* (10 min) to remove cellular debris. The supernatant was dried and dissolved in sterile water. Determination of the dNTP pool size was based on DNA polymerase-catalyzed incorporation of radioactive dNTP into synthetic oligonucleotide templates as described (Sherman and Fyfe, 1989).

### Xenograft Studies

Mice were given food and water ad libitum, and all animal procedures were carried out under a project license issued by the home office (London, United Kingdom) following ethical review by the University of Oxford Committee on Animal Care, and performed according to United Kingdom Coordinating Committee on Cancer Research guidelines. BALB/C nude mice (female, 6–8 weeks old) were injected subcutaneously with A498, LB996, or U2OS tumor cells (5 × 10^6^ cells per mouse in 50% v/v Matrigel, 14 mice for A498 and U2OS, 10 for LB996). When the mean tumor size reached 110 mm^3^, mice were divided into two equal groups. Group 1 received vehicle alone (0.5% w/v methylcellulose, 0.1 ml/10 g body weight, twice daily by oral gavage) for 12 days. Group 2 received AZD1775 (60 mg/kg in 0.5% w/v methylcellulose, 0.1 ml/10 g body weight, twice daily by oral gavage) for 12 days. Tumor size was determined by caliper measurements and calculated as (width × length × height) /2. All mice in the control group were killed on day 13, 24 hr after the last dose of vehicle. Three mice in the treatment group were killed on day 13, 24 hr after the last dose of AZD1775, and tumor re-growth in any remaining mice was followed until day 33, when all remaining mice were killed.

### Immunohistochemistry on Tissue Microarray

Use of human tissues in research was approved by National Research Ethics Service Committee Oxfordshire REC C (study 07/H0606/120). Nephrectomies were collected between 1995 and 2004, with informed consent from all subjects to use of tissue in ethically approved research. Formalin-fixed paraffin-embedded tumor cores were used to construct renal cancer tissue microarrays, and 4 μm sections were stained on Bondmax Autostainer (Leica). Briefly, following heat-induced epitope retrieval (20 min, pH 9) sections were stained with an antibody against H3K36me3 (Mab-183-050, Diagenode) at 1:1,000 dilution for 1 hr. Antibody binding was identified using a Polymer Detection System (Leica), followed by 3,3′-diaminobenzidine staining.

## Author Contributions

E.M., Y.J., and S.S. are co-second authors for this paper, having contributed equally to the paper. S.X.P., E.M., Y.J., S.S., M.W., L.P.Z., G.O., I.M., and N.D. designed and conducted the experiments with contributions from C.C.P.; C.V. and V.M. were involved in patient tissue microarray studies. S.X.P. and T.C.H. wrote the manuscript. G.D., S.Y., N.B.L.T., V.A., A.R., and T.C.H. supervised the work. Figure 1 was obtained by S.X.P.; Figure 2 was obtained by S.X.P., E.M., and M.W.; Figure 3 was obtained by S.X.P., S.S., E.M., G.O., and L.P.Z.; Figure 4 was obtained by S.X.P., I.M., G.O., and E.M.; Figure 5 was obtained by Y.J. and N.D.

## Supplemental Information

Document S1. Supplemental Experimental Procedures and Figures S1–S5

Document S2. Article plus Supplemental Information
