# Supplementary material for: Inhibiting WEE1 Selectively Kills Histone H3K36me3-Deficient Cancers by dNTP Starvation
Source: Cancer Cell. 2015 Nov 9;28(5):557–68. doi: 10.1016/j.ccell.2015.09.015 (PMC4643307; doi:10.1016/j.ccell.2015.09.015)
Supplement: Document S1. Supplemental Experimental Procedures and Figures S1–S5 [file mmc1.pdf]

Cancer Cell, Volume *28*

Supplemental Information

Inhibiting WEE1 Selectively Kills Histone H3K36me3-Deficient Cancers by dNTP Starvation

Sophia X. Pfister, Enni Markkanen, Yanyan Jiang, Sovan Sarkar, Mick Woodcock, Giulia Orlando, Ioanna Mavrommati, Chen-Chun Pai, Lykourgos-Panagiotis Zalmas, Neele Drobnitzky, Grigory L. Dianov, Clare Verrill, Valentine M. Macaulay, Songmin Ying, Nicholas B. La Thangue, Vincenzo D'Angiolella, Anderson J. Ryan, and Timothy C. Humphrey

Supplemental Data

A

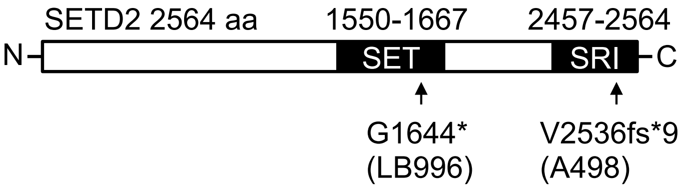

B

Predicted off-target site #1

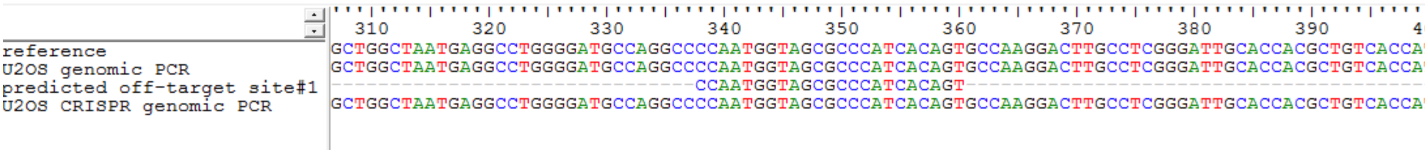

Predicted off-target site #2

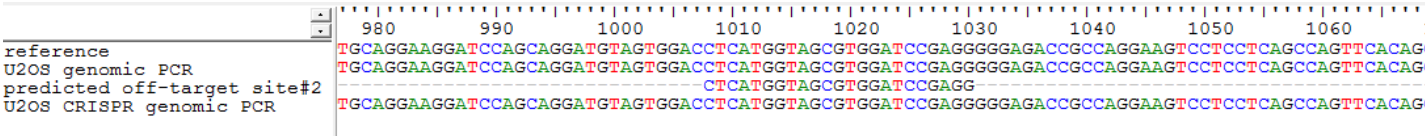

Predicted off-target site #3

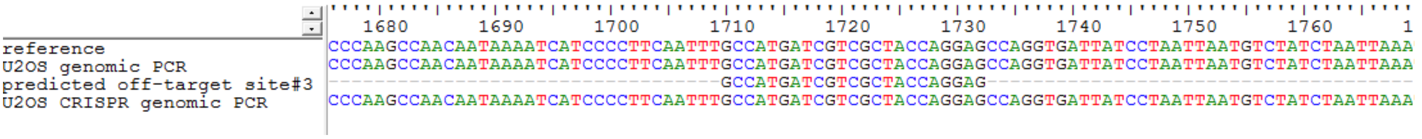

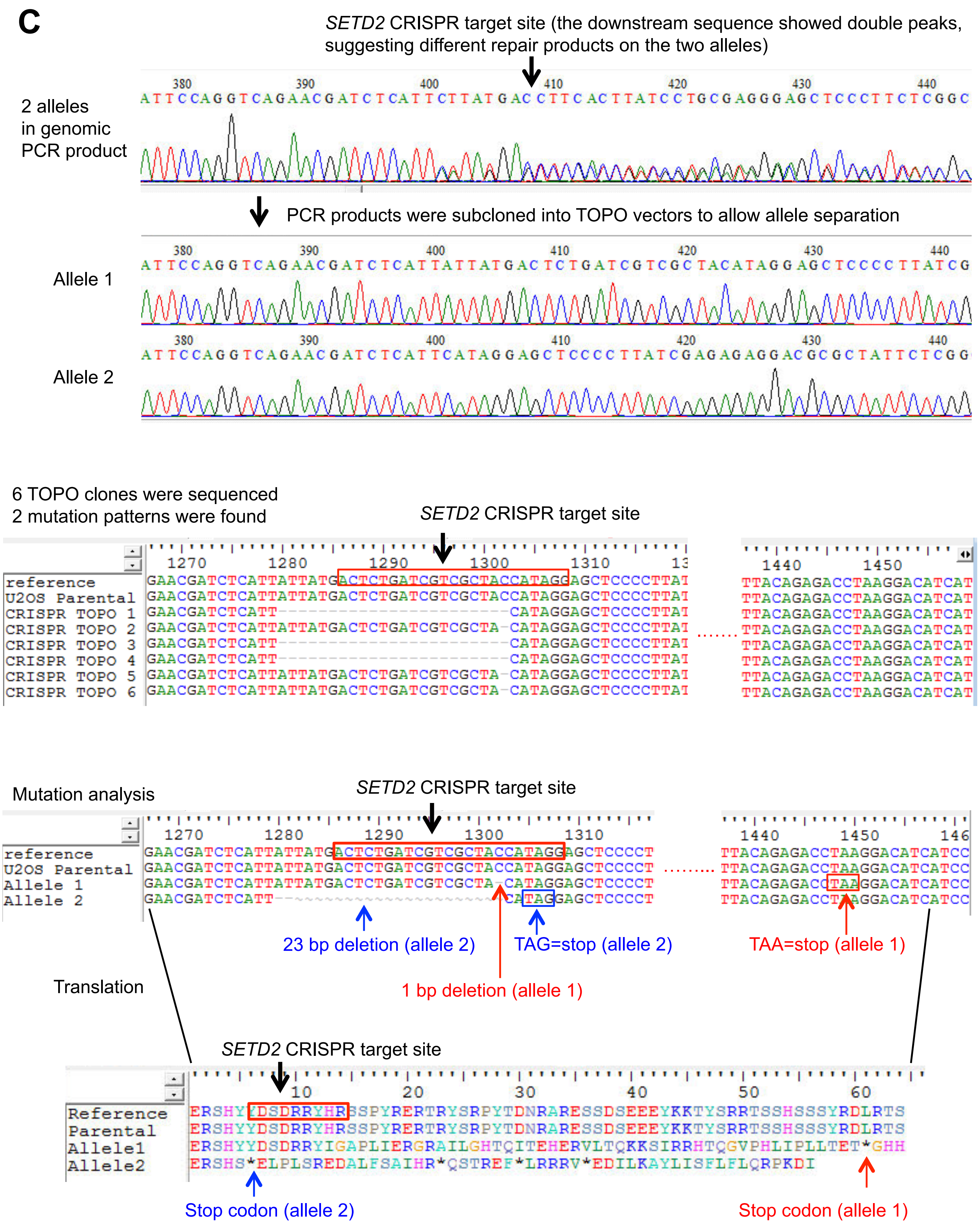

C
SETD2 CRISPR target site (the downstream sequence showed double peaks, suggesting different repair products on the two alleles)
2 alleles in genomic PCR product
380 390 400 410 420 430 440
ATTCCAGGTCAGAACGATCTCATTCTTATGACCTTCACTTATCCTGCGAGGGAGCTCCCTTCTCGGC
PCR products were subcloned into TOPO vectors to allow allele separation
Allele 1
ATTCCAGGTCAGAACGATCTCATTATTATGACTCTGATCGTCGCTACATAGGAGCTCCCCTTATCG
Allele 2
ATTCCAGGTCAGAACGATCTCATTCATAGGAGCTCCCCTTATCGAGAGAGGACGCGCTATTCTCGG
6 TOPO clones were sequenced
2 mutation patterns were found
SETD2 CRISPR target site
1270 1280 1290 1300 1310 1440 1450
reference GAACGATCTCATTATTATGACTCTGATCGTCGCTACCATAGGAGCTCCCCTTAT TTACAGAGACCTAAGGACATCAT
U2OS Parental GAACGATCTCATTATTATGACTCTGATCGTCGCTACCATAGGAGCTCCCCTTAT TTACAGAGACCTAAGGACATCAT
CRISPR TOPO 1 GAACGATCTCATT-----CATAGGAGCTCCCCTTAT TTACAGAGACCTAAGGACATCAT
CRISPR TOPO 2 GAACGATCTCATTATTATGACTCTGATCGTCGCTA-CATAGGAGCTCCCCTTAT TTACAGAGACCTAAGGACATCAT
CRISPR TOPO 3 GAACGATCTCATT-----CATAGGAGCTCCCCTTAT TTACAGAGACCTAAGGACATCAT
CRISPR TOPO 4 GAACGATCTCATT-----CATAGGAGCTCCCCTTAT TTACAGAGACCTAAGGACATCAT
CRISPR TOPO 5 GAACGATCTCATTATTATGACTCTGATCGTCGCTA-CATAGGAGCTCCCCTTAT TTACAGAGACCTAAGGACATCAT
CRISPR TOPO 6 GAACGATCTCATTATTATGACTCTGATCGTCGCTA-CATAGGAGCTCCCCTTAT TTACAGAGACCTAAGGACATCAT
Mutation analysis
SETD2 CRISPR target site
reference GAACGATCTCATTATTATGACTCTGATCGTCGCTACCATAGGAGCTCCCCT TTACAGAGACCTAAGGACATCATCC
U2OS Parental GAACGATCTCATTATTATGACTCTGATCGTCGCTACCATAGGAGCTCCCCT TTACAGAGACCTAAGGACATCATCC
Allele 1 GAACGATCTCATTATTATGACTCTGATCGTCGCTA-CATAGGAGCTCCCCT TTACAGAGACCTAAGGACATCATCC
Allele 2 GAACGATCTCATT-----CATAGGAGCTCCCCT TTACAGAGACCTAAGGACATCATCC
23 bp deletion (allele 2)
TAG=stop (allele 2)
TAA=stop (allele 1)
1 bp deletion (allele 1)
Translation
SETD2 CRISPR target site
10 20 30 40 50 60
Reference ERSHYYDSDRRYHRSSPYRERTRYSRPYTDNRARESSDSEEEYKKTYSRRTSSHSSSYRDLRTS
Parental ERSHYYDSDRRYHRSSPYRERTRYSRPYTDNRARESSDSEEEYKKTYSRRTSSHSSSYRDLRTS
Allele1 ERSHYYDSDRRYIGAPLIERGRAILGHTQITEHERVLTQKKSIRRHTQGVPHLIPLLTET*GHH
Allele2 ERSHS*ELPLSREDALFSAIHR*QSTREF*LRRRV*EDILKAYLISFLFLQRPKDI
Stop codon (allele 2)
Stop codon (allele 1)

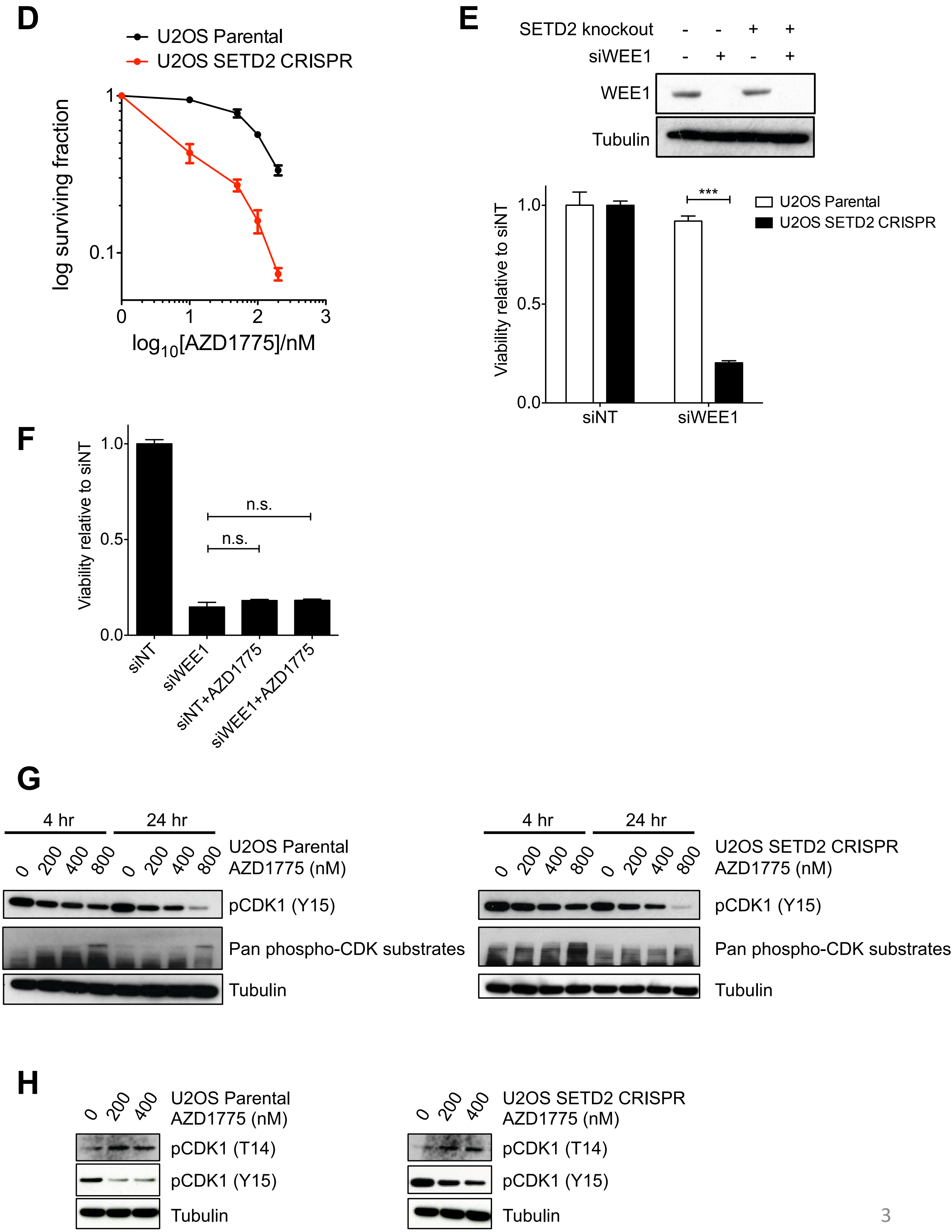
D
U2OS Parental
U2OS SETD2 CRISPR
log surviving fraction
1
0.1
0
1
2
3
log10[AZD1775]/nM
E
SETD2 knockout - - + +
siWEE1 - + - +
WEE1
Tubulin
Viability relative to siNT
1.0
0.5
0.0
***
U2OS Parental
U2OS SETD2 CRISPR
siNT
siWEE1
F
Viability relative to siNT
1.0
0.5
0.0
n.s.
n.s.
siNT
siWEE1
siNT+AZD1775
siWEE1+AZD1775
G
4 hr
24 hr
0 200 400 800 0 200 400 800
U2OS Parental
AZD1775 (nM)
pCDK1 (Y15)
Pan phospho-CDK substrates
Tubulin
4 hr
24 hr
0 200 400 800 0 200 400 800
U2OS SETD2 CRISPR
AZD1775 (nM)
pCDK1 (Y15)
Pan phospho-CDK substrates
Tubulin
H
0 200 400
U2OS Parental
AZD1775 (nM)
pCDK1 (T14)
pCDK1 (Y15)
Tubulin
0 200 400
U2OS SETD2 CRISPR
AZD1775 (nM)
pCDK1 (T14)
pCDK1 (Y15)
Tubulin

Figure S1, related to Figure 1. H3K36me3-deficient cells are hypersensitive to WEE1 inhibition.

(A) Schematic map showing *SETD2* mutations in LB996 and A498 cell lines. The locations of SET domain and SRI domain were mapped according to Uniprot.org. The amino acid changes are quoted from published literature but adjusted to the updated sequence of SETD2 (in the new version, 503 amino acids were added to the N-terminus of the protein). A498 expresses a non-functional SETD2 protein while LB996 does not express SETD2 protein.

(B) Analysis of potential off-target *SETD2*-CRISPR sites. Potential off-target sites of the gRNA was predicted using the MIT CRISPR design tool (http://crispr.mit.edu/). The top three predicted off-target sites were chosen. Genomic DNA was extracted from U2OS parental and U2OS CRISPR SETD2-knockout cells. PCR was performed on genomic DNA with primers located approximately 500 bp on each side of the predicted off-target site. The PCR product was cleaned by Qiagen spin column and sequenced by Sanger sequencing. The sequences were aligned using BioEdit ClustalW multiple sequence alignment tool.

(C) Validation of CRISPR SETD2-knockout. To study the mutation patterns on the two alleles in the CRISPR knockout cell, genomic DNA was extracted from U2OS parental and U2OS CRISPR SETD2-knockout cells. Genomic PCR was performed as in (B) with primers 500 bp from the target site, and the PCR products were subcloned into a TOPO vector, transformed into competent cells, and 6 clones were sequenced separately by Sanger sequencing. The sequences were aligned using BioEdit ClustalW multiple sequence alignment tool and the amino acid translation was cross-checked with the amino acid sequence from CCDS report.

(D) Clonogenic survival curves of U2OS parental and U2OS CRISPR SETD2-knockout cells after exposure to WEE1 inhibitor AZD1775 (4 days) at indicated concentrations. Colonies were allowed to form for 14 days. Data are presented as mean ± SEM, n = 3 independent experiments.

(E) U2OS or U2OS CRISPR SETD2-knockout cells were transfected with either control siRNA (siNT) or WEE1 siRNA (siWEE1) for 5 days, viability was measured by Resazurin. Data are presented as mean ± SEM, n = 3 independent experiments. ***$p < 0.001$. Western blot showing WEE1 protein levels 48 hr after siRNA transfection.

(F) U2OS CRISPR SETD2-knockout cells were transfected with siNT or siWEE1 for 48 hr before treatment with either DMSO or AZD1775 (400 nM). Viability was measured by Resazurin 3 days after inhibitor treatment. Data are presented as mean ± SEM, n = 3 independent experiments. n.s. not significant.

(G) Western blot analysis of pCDK1 (Y15) and pan-CDK substrate levels in U2OS and U2OS CRISPR SETD2-knockout cells. Cells were treated with indicated concentrations of AZD1775 and harvested at the indicated times. Lysis buffer contained protease inhibitor and phosphatase inhibitor cocktail. Western blots were performed using anti-phospho-CDK1 (Tyr 15) or anti-phospho-pan-CDK antibodies. Tubulin was used as loading control.

(H) Western blot analysis of pCDK1 T14 and Y15 levels in U2OS and U2OS CRISPR SETD2-knockout cells. Cells were treated with indicated concentrations of AZD1775 and harvested after 24 hr. Western blots were performed using antibodies against phospho-CDK1 (Thr 14) (substrate of MYT1) and phospho-CDK1 (Tyr 15) (substrate of WEE1). Tubulin was used as loading control.

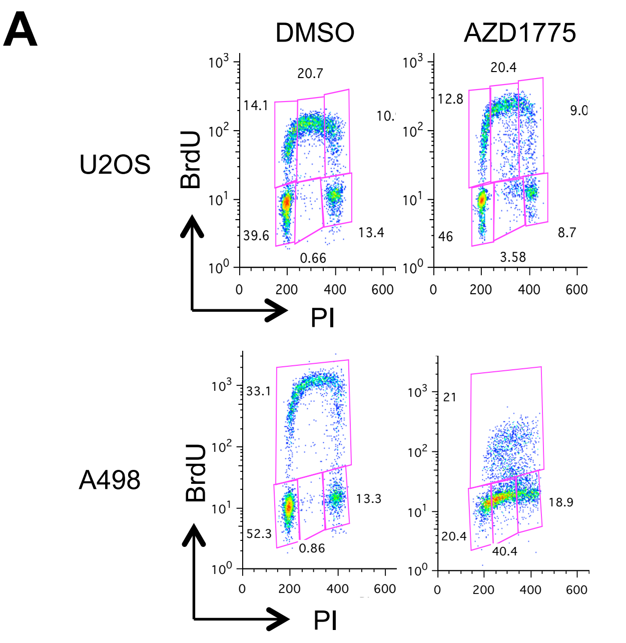

Non-replicating S	DMSO	AZD1775
U2OS	0.7%	3.6%
A498	0.9%	40.4%

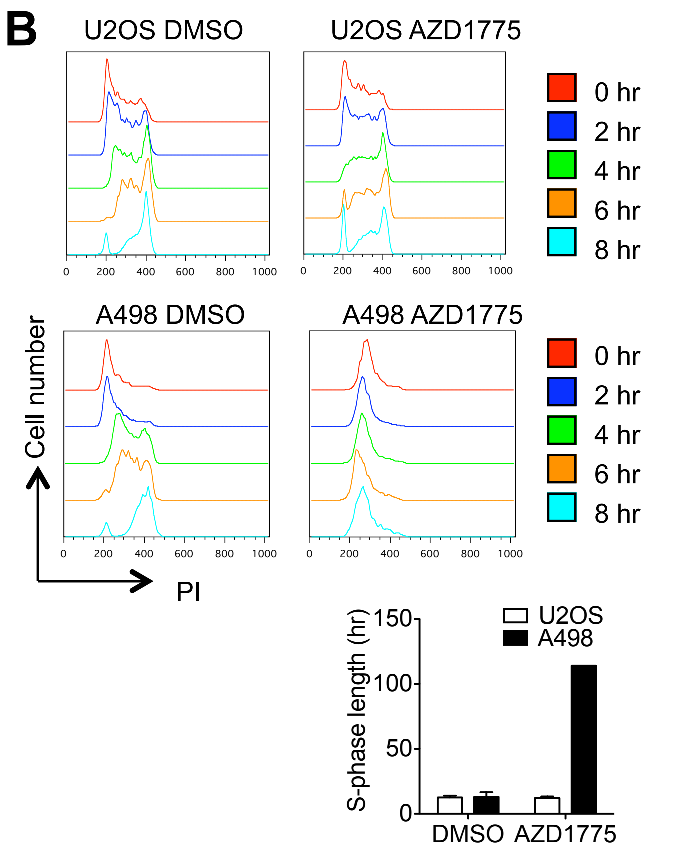

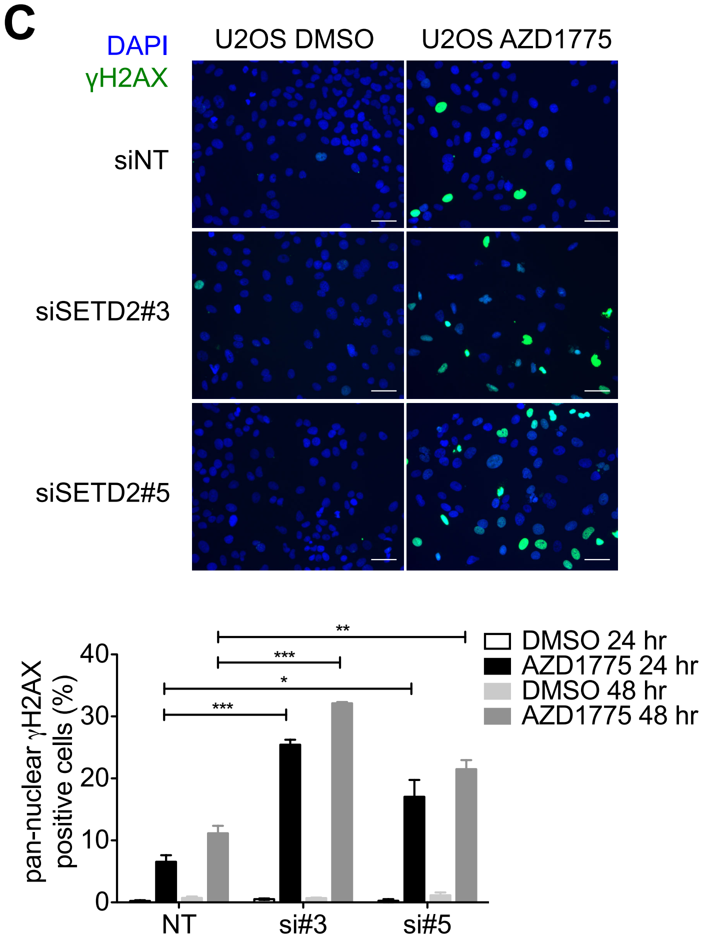

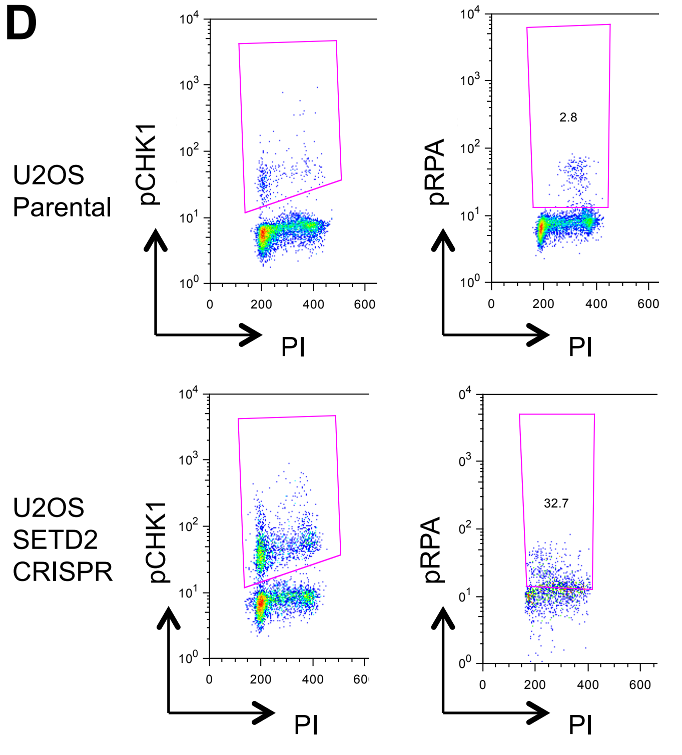

AZD1775	pCHK1	pRPA
U2OS parental	4.6%	2.8%
U2OS SETD2 CRISPR	33.2%	32.7%

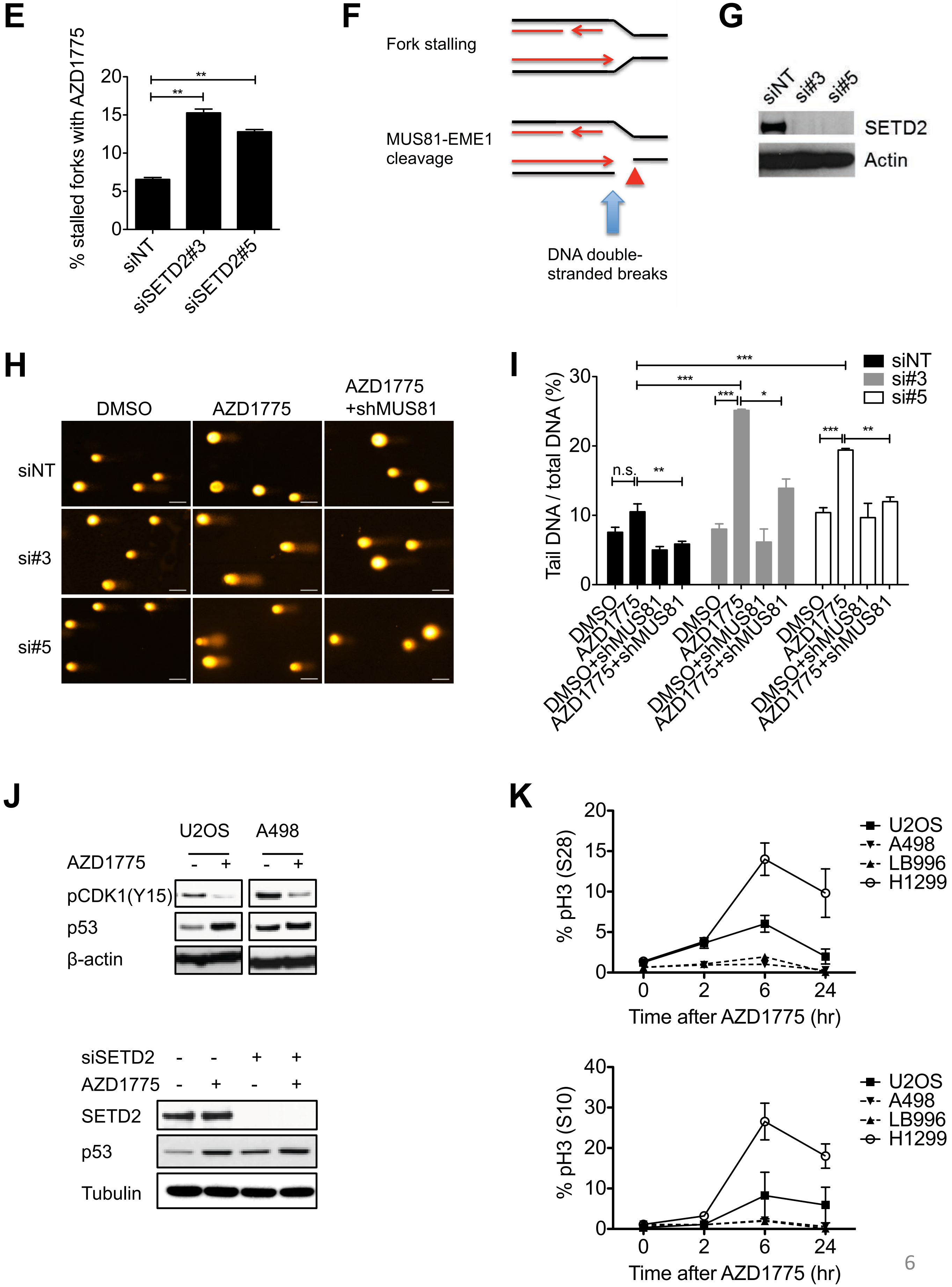
E
% stalled forks with AZD1775
20
15
10
5
0
**
**
siNT
siSETD2#3
siSETD2#5
F
Fork stalling
MUS81-EME1 cleavage
DNA double-stranded breaks
G
siNT
si#3
si#5
SETD2
Actin
H
DMSO
AZD1775
AZD1775 +shMUS81
siNT
si#3
si#5
I
Tail DNA / total DNA (%)
30
20
10
0
***
***
***
*
***
**
n.s.
**
siNT
si#3
si#5
DMSO
AZD1775
DMSO+shMUS81
AZD1775+shMUS81
J
U2OS
A498
AZD1775
- + - +
pCDK1(Y15)
p53
β-actin
siSETD2 - - + +
AZD1775 - + - +
SETD2
p53
Tubulin
K
% pH3 (S28)
20
15
10
5
0
U2OS
A498
LB996
H1299
0
2
6
24
Time after AZD1775 (hr)
% pH3 (S10)
40
30
20
10
0
U2OS
A498
LB996
H1299
Time after AZD1775 (hr)

L

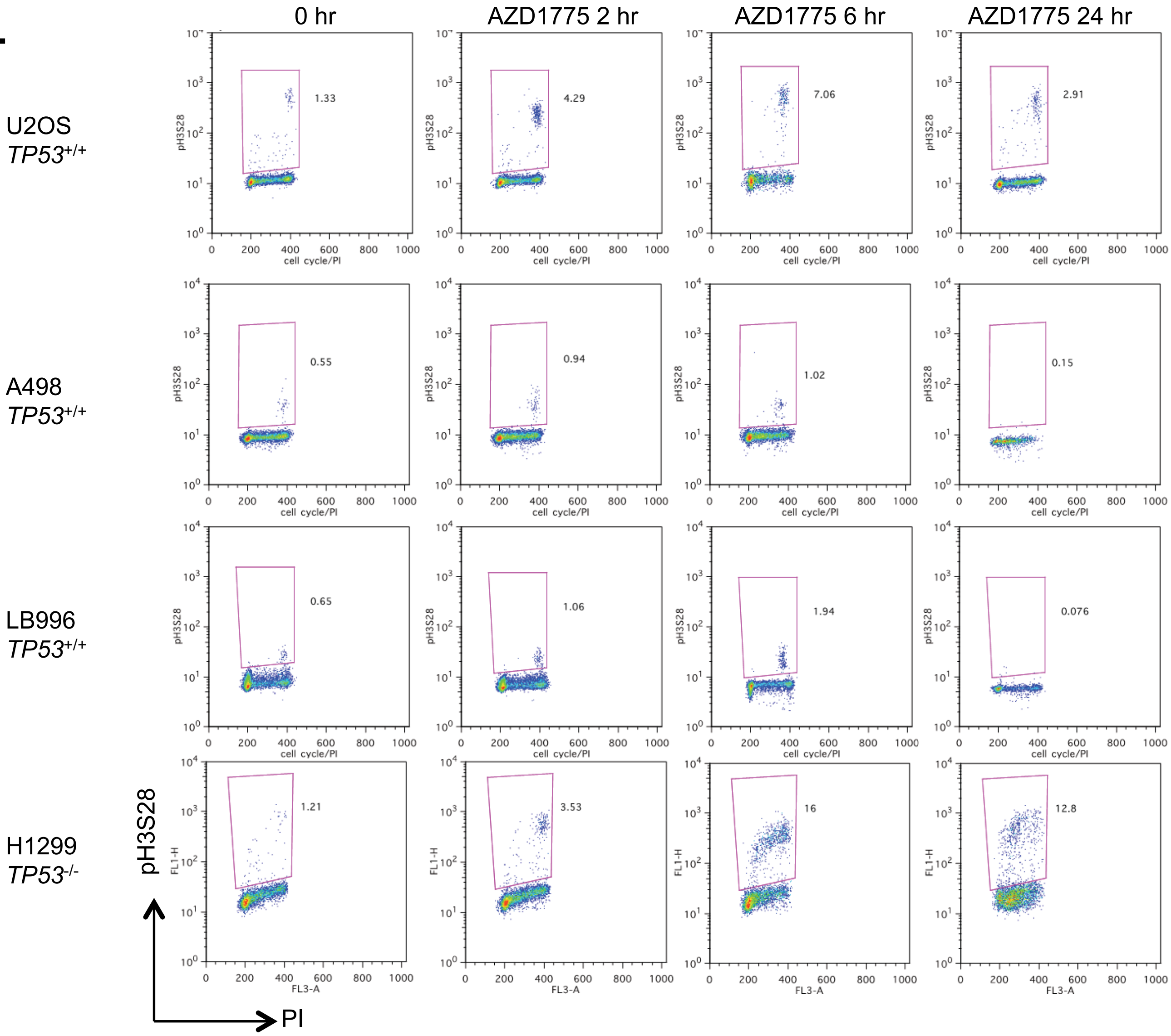

M

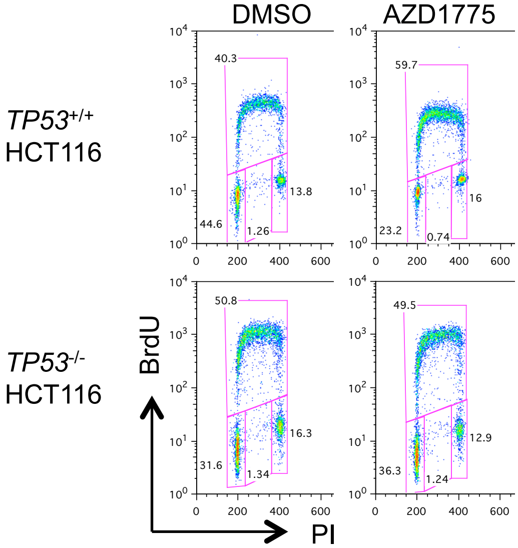

N

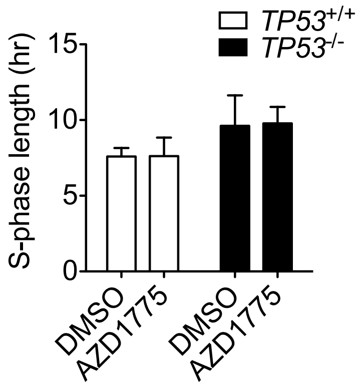

Non-replicating S	DMSO	AZD1775
HCT116 *TP53*$^{+/+}$	1.3%	0.7%
HCT116 *TP53*$^{-/-}$	1.3%	1.2%

O

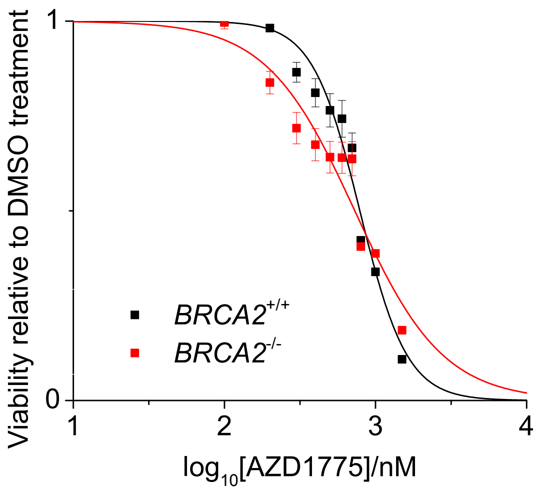

Viability relative to DMSO treatment
log10[AZD1775]/nM
BRCA2+/+
BRCA2-/-

P

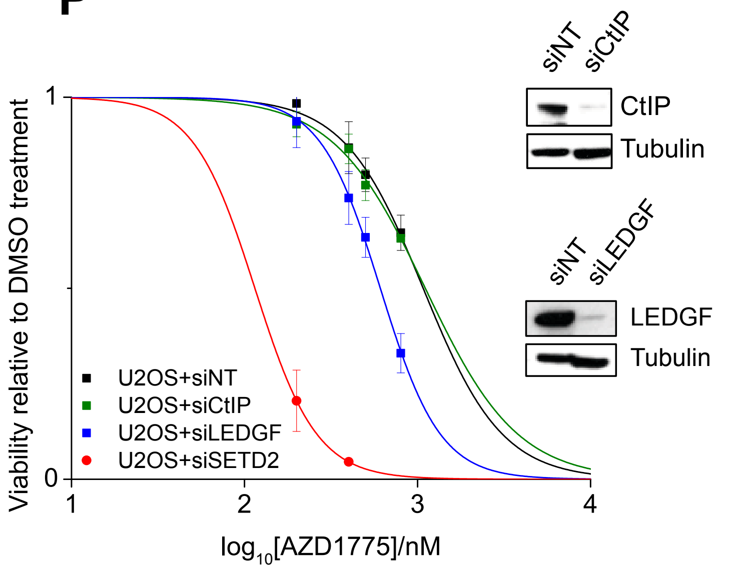

Viability relative to DMSO treatment
log10[AZD1775]/nM
U2OS+siNT
U2OS+siCtIP
U2OS+siLEDGF
U2OS+siSETD2
siNT
siCtIP
CtIP
Tubulin
siNT
siLEDGF
LEDGF
Tubulin

Q

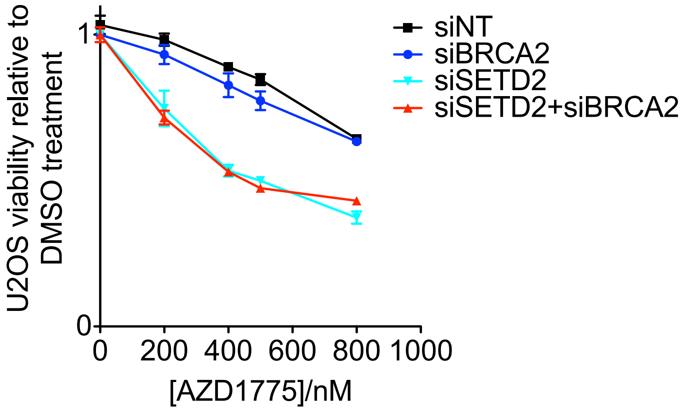

U2OS viability relative to DMSO treatment
[AZD1775]/nM
siNT
siBRCA2
siSETD2
siSETD2+siBRCA2

R

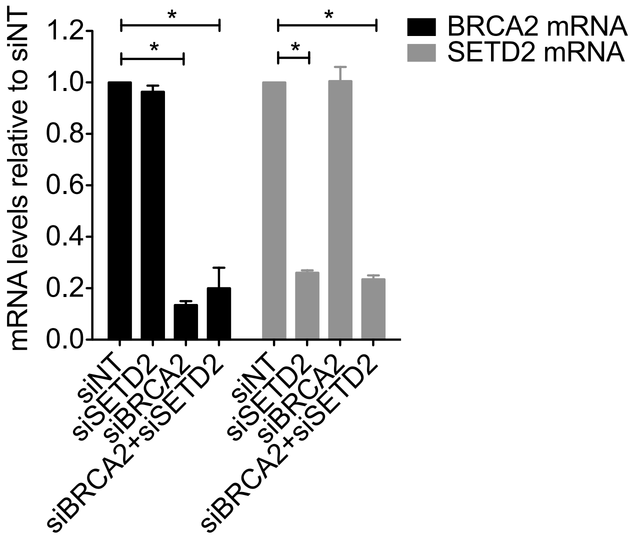

mRNA levels relative to siNT
BRCA2 mRNA
SETD2 mRNA
siNT
siSETD2
siBRCA2
siBRCA2+siSETD2

Figure S2, related to Figure 2. WEE1 inhibitor induces replication stress in SETD2-deficient cells.

(A) Cell cycle analysis of SETD2 wild-type (U2OS) or SETD2-deficient (A498) cells after treatment with WEE1 inhibitor AZD1775 (200 nM) for 48 hr. Percent non-replicating S-phase population is indicated.

(B) U2OS and A498 cells were treated with DMSO or AZD1775 (200 nM) (48 hr) and pulse-labeled with BrdU for 20 min. Cell cycle progression of the BrdU-labeled (S-phase) population was monitored at times indicated. The time for cells to complete S-phase (S-phase length) was calculated according to Begg et al., 1985.

(C) U2OS cells were transfected with control siRNA (siNT) or SETD2 siRNAs (si#3 and si#5) 48 hr prior to DMSO or AZD1775 (200 nM) treatment for 24 or 48 hr. Representative immunofluorescence microscope images of γH2AX staining at 24 hr were shown. Scale bar = 50 μM. Percentage of cells with pan-nuclear staining was analyzed by InCell Analyzer.

(D) U2OS or U2OS SETD2 CRISPR cells were treated with AZD1775 (200 nM) for 48 hr. Percentage of cells with positive staining for phospho-CHK1 (S345) and phospho-RPA (S33) was analyzed by FACS.

(E) DNA fiber analysis of stalled replication forks (tracks with 1st label only) in U2OS cells transfected with either non-targeting siRNA (siNT) or SETD2 siRNAs (si#3 and si#5) (48 hr) prior to AZD1775 (200 nM) (48 hr).

(F) Schematic map showing MUS81 cleavage of stalled replication forks.

(G) Western blot showing SETD2 protein levels after SETD2 siRNA treatment (si#3 and si#5) in the comet assay in (H) and (I).

(H) Alkaline comet assay of U2OS cells transfected with control siRNA (siNT) or SETD2 siRNA (si#3 and si#5) and MUS81 shRNA lentiviral particles 48 hr prior to treatment with DMSO or AZD1775 (200 nM) for 48 hr. Representative images of nuclear DNA after electrophoresis. Scale bar = 50 μM.

(I) Quantification of the levels of DNA damage in U2OS cells from the comet assay in (H), presented as the percentage of DNA in the tail over total DNA.

(J) Western blots showing the levels of CDK1 inhibitory phosphorylation at Tyrosine 15 (Y15), and p53 stabilization in U2OS, A498 (top) or U2OS SETD2-knockdown cells (bottom) after treatment with the WEE1 inhibitor AZD1775 (200 nM) for 48 hr.

(K) Quantification of H3 phosphorylation at Serine 28 or Serine 10 (both are mitotic markers) in $TP53^{+/+}$ cells (U2OS, A498, LB996) and $TP53^{-/-}$ cells (H1299) at indicated time after AZD1775 (200 nM) treatment, as measure by FACS.

(L) FACS analysis of phospho-histone H3S28 at indicated time after AZD1775 (200 nM) treatment.

(M) FACS analysis of BrdU incorporation by $TP53^{+/+}$ or $TP53^{-/-}$ HCT116 cells after treatment with DMSO or AZD1775 (200 nM) (48 hr).

(N) Estimated S-phase length in isogenic $TP53^{+/+}$ or $TP53^{-/-}$ HCT116 cells treated with DMSO or AZD1775, calculated according to an established protocol (Begg et al., 1985).

(O) Viability of $BRCA2^{+/+}$ and $BRCA2^{-/-}$ isogenic DLD1 cell lines measured by Resazurin 5 days after a single treatment with WEE1 inhibitor AZD1775 at the indicated concentrations.

(P) Viability of U2OS cells transfected with siRNAs against LEDGF, CtIP or SETD2. 48 hr after transfection, cells were treated with AZD1775 and viability was measured by Resazurin after 3 days.

(Q) Viability of U2OS cells transfected with siRNAs against SETD2, BRCA2 or SETD2+BRCA2. 48 hr after transfection, cells were treated with AZD1775 and viability was measured by Resazurin after 3 days.

(R) qRT-PCR analysis of SETD2 and BRCA2 mRNA levels in (Q).

All data in Figure S2 are presented as mean ± SEM, n = 3 independent experiments. Unpaired, two-tailed t-test was performed, ***p < 0.001, **p < 0.01, *p < 0.05, n.s. not significant.

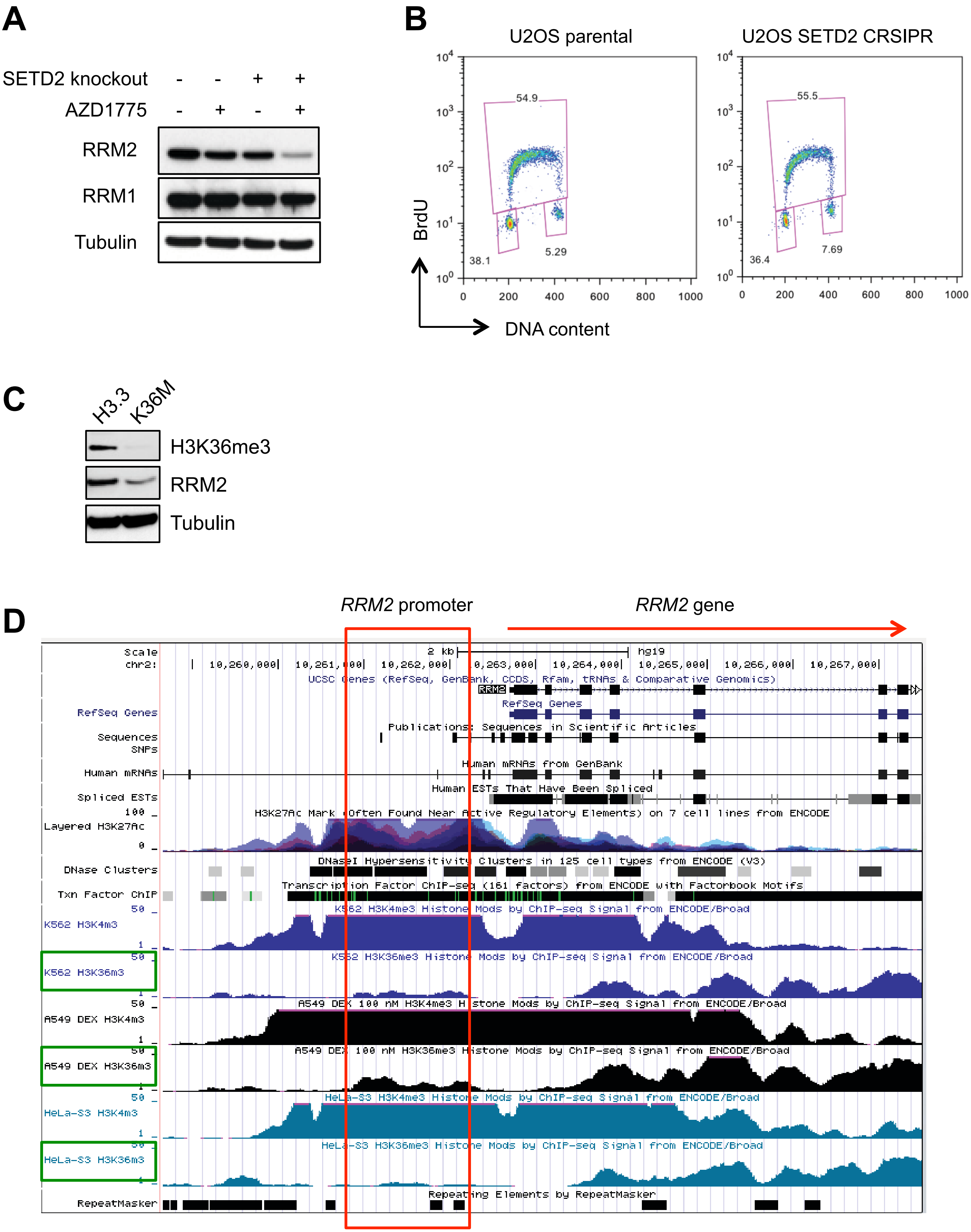
A
SETD2 knockout - - + +
AZD1775 - + - +
RRM2
RRM1
Tubulin
B
U2OS parental
U2OS SETD2 CRSIPR
54.9
38.1
5.29
55.5
36.4
7.69
BrdU
DNA content
0
200
400
600
800
1000
C
H3.3
K36M
H3K36me3
RRM2
Tubulin
D
RRM2 promoter
RRM2 gene
Scale
chr2:
2 kb
hg19
10,260,000
10,261,000
10,262,000
10,263,000
10,264,000
10,265,000
10,266,000
10,267,000
UCSC Genes (RefSeq, GenBank, CCDS, Rfam, tRNAs & Comparative Genomics)
RRM2
RefSeq Genes
Publications: Sequences in Scientific Articles
Sequences
SNPs
Human mRNAs from GenBank
Human mRNAs
Human ESTs That Have Been Spliced
Spliced ESTs
H3K27Ac Mark (Often Found Near Active Regulatory Elements) on 7 cell lines from ENCODE
Layered H3K27Ac
DNaseI Hypersensitivity Clusters in 125 cell types from ENCODE (V3)
DNase Clusters
Transcription Factor ChIP-seq (161 factors) from ENCODE with Factorbook Motifs
Txn Factor ChIP
K562 H3K4me3 Histone Mods by ChIP-seq Signal from ENCODE/Broad
K562 H3K4m3
K562 H3K36me3 Histone Mods by ChIP-seq Signal from ENCODE/Broad
K562 H3K36m3
A549 DEX 100 nM H3K4me3 Histone Mods by ChIP-seq Signal from ENCODE/Broad
A549 DEX H3K4m3
A549 DEX 100 nM H3K36me3 Histone Mods by ChIP-seq Signal from ENCODE/Broad
A549 DEX H3K36m3
HeLa-S3 H3K4me3 Histone Mods by ChIP-seq Signal from ENCODE/Broad
HeLa-S3 H3K4m3
HeLa-S3 H3K36me3 Histone Mods by ChIP-seq Signal from ENCODE/Broad
HeLa-S3 H3K36m3
Repeating Elements by RepeatMasker
RepeatMasker

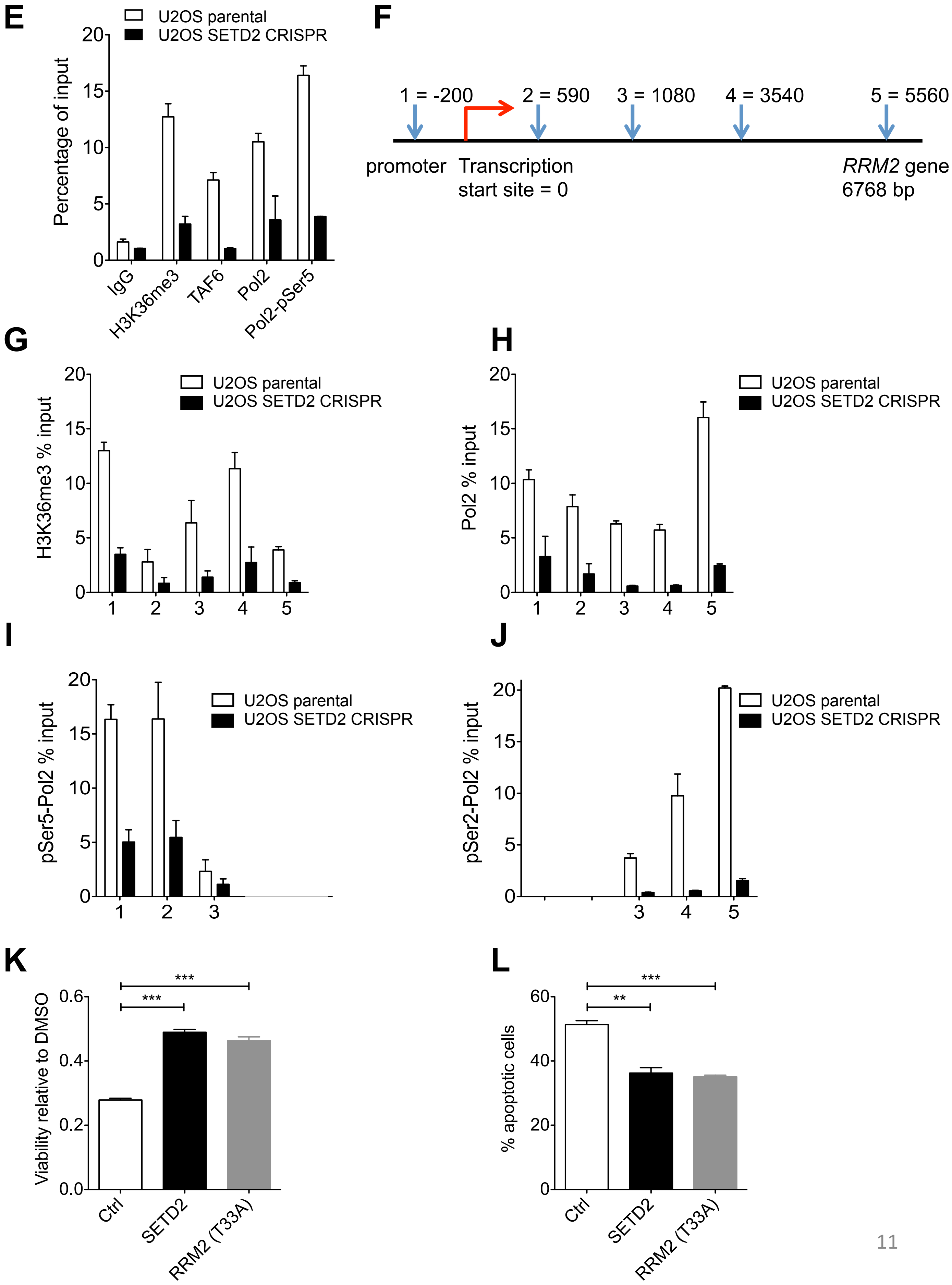
E
U2OS parental
U2OS SETD2 CRISPR
Percentage of input
0
5
10
15
20
IgG
H3K36me3
TAF6
Pol2
Pol2-pSer5
F
1 = -200
2 = 590
3 = 1080
4 = 3540
5 = 5560
promoter
Transcription start site = 0
RRM2 gene 6768 bp
G
U2OS parental
U2OS SETD2 CRISPR
H3K36me3 % input
0
5
10
15
20
1
2
3
4
5
H
U2OS parental
U2OS SETD2 CRISPR
Pol2 % input
0
5
10
15
20
1
2
3
4
5
I
U2OS parental
U2OS SETD2 CRISPR
pSer5-Pol2 % input
0
5
10
15
20
1
2
3
J
U2OS parental
U2OS SETD2 CRISPR
pSer2-Pol2 % input
0
5
10
15
20
3
4
5
K
***
***
Viability relative to DMSO
0.0
0.2
0.4
0.6
Ctrl
SETD2
RRM2 (T33A)
L
***
**
% apoptotic cells
0
20
40
60
Ctrl
SETD2
RRM2 (T33A)

Figure S3, related to Figure 3. H3K36me3 facilitates *RRM2* transcription.

(A) Western blot analysis of RRM2 and RRM1 levels in U2OS and U2OS CRISPR SETD2-knockout cells after exposure to either DMSO or AZD1775 (200 nM) (24 hr).

(B) FACS cell cycle analysis of BrdU incorporation by U2OS parental and U2OS CRISPR SETD2-knockout cells without any treatment.

(C) Western blot analysis of H3K36me3 and RRM2 protein levels in U2OS cells expressing either a dominant-negative H3.3K36M transgene (K36M) or the control H3.3 gene (H3.3).

(D) ENCODE database analysis of the co-localization of H3K36me3 with H3K4me3 (a mark for transcription initiation) at the promoter of the *RRM2* gene. The red window indicates the promoter region of *RRM2*. ChIP-seq data from three cancer cell lines (K562, HeLa and A549) are presented.

(E) ChIP-qPCR analysis of the enrichment of H3K36me3, TAF6, RNA-Pol2 and phosphor-Ser5 Pol2 at the promoter of the *RRM2* gene. U2OS parental cells or U2OS CRISPR SETD2-knockout cells were harvested 24 hr after seeding.

(F) Schematic map showing the qPCR primer locations on the *RRM2* gene used to amplify the chromatin after ChIP (in G-J).

(G) ChIP-qPCR analysis of the enrichment of H3K36me3 across the gene body of *RRM2*, using primers described in (F).

(H) ChIP-qPCR analysis of the enrichment of RNA polymerase 2 (Pol2) across the gene body of *RRM2*, using primers described in (F).

(I) ChIP-qPCR analysis of enrichment of phospho-Pol2 at serine 5 (pSer5-pol2) at primer positions 1-3 of the *RRM2* gene, using primers described in (F).

(J) ChIP-qPCR analysis of enrichment of phospho-Pol2 at serine 2 (pSer2-pol2) at primer positions 3-5 of the *RRM2* gene, using primers described in (F). (pSer5-pol2 is usually increased at the 5' end of the transcribed gene while pSer2-pol2 increases towards the 3' end of the gene.) U2OS parental cells or U2OS SETD2 CRISPR knockout cells were harvested 24 hr after seeding.

(K) Relative viability of U2OS CRISPR SETD2-knockout cells after transient transfection with control plasmid, plasmid containing *SETD2* cDNA or plasmid containing *RRM2 (T33A)* cDNA. 48 hr after transfection, cells were treated with DMSO or AZD1775 and cell viability measured by Resazurin after 3 days. Viability was normalised to DMSO treatment in each condition.

(L) U2OS CRISPR SETD2-knockout cells were transiently transfected as in (K). Percentage of apoptotic cells was measured 48 hr after AZD1775 treatment.

All data in Figure S3 are presented as mean ± SEM, n = 3 independent experiments. Unpaired, two-tailed t-test was performed, ***$p < 0.001$, **$p < 0.01$, *$p < 0.05$.

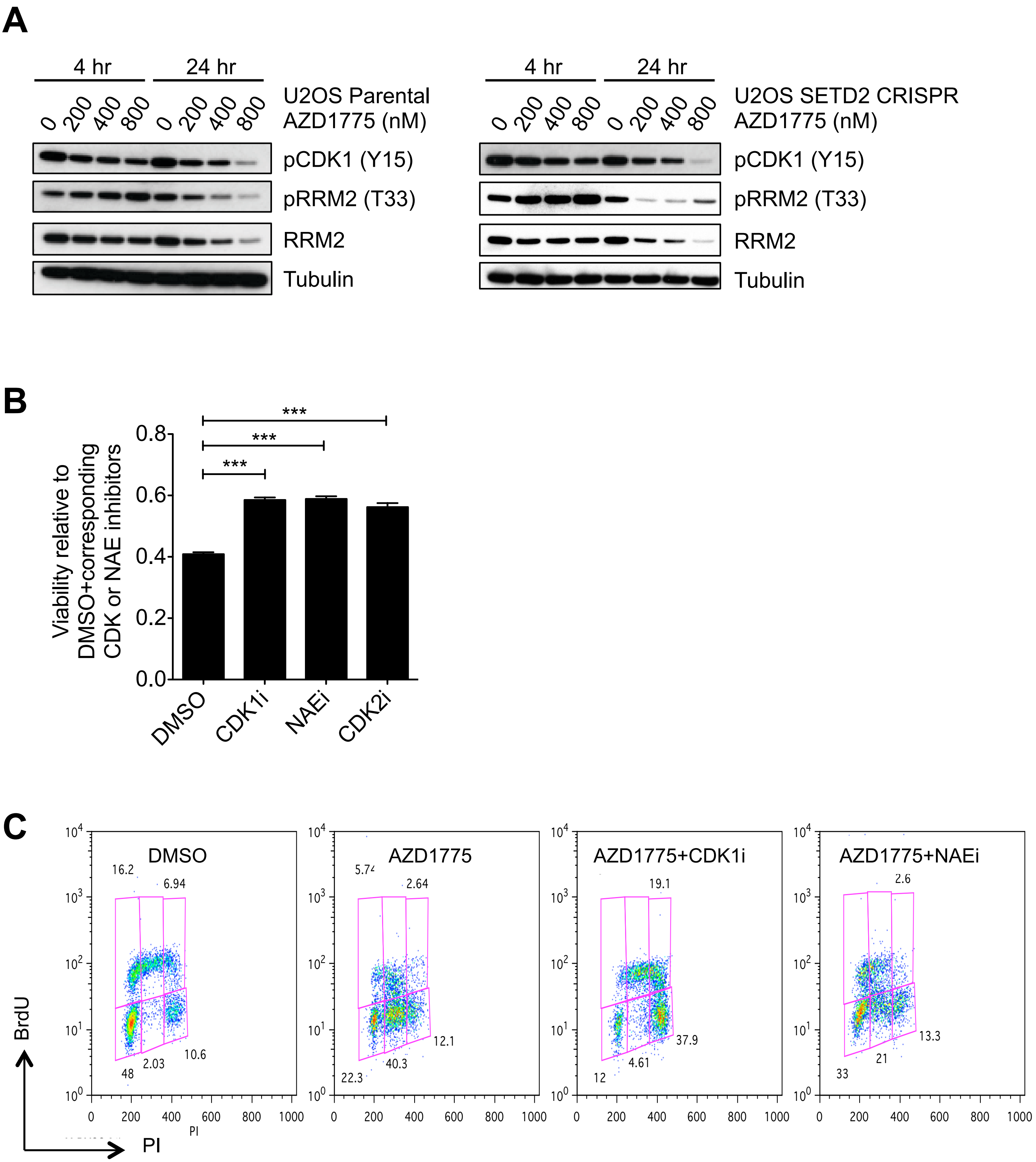

	DMSO	AZD1775	AZD1775+CDK1i	AZD1775+NAEi
Non-replicating S	2%	40%	5%	21%

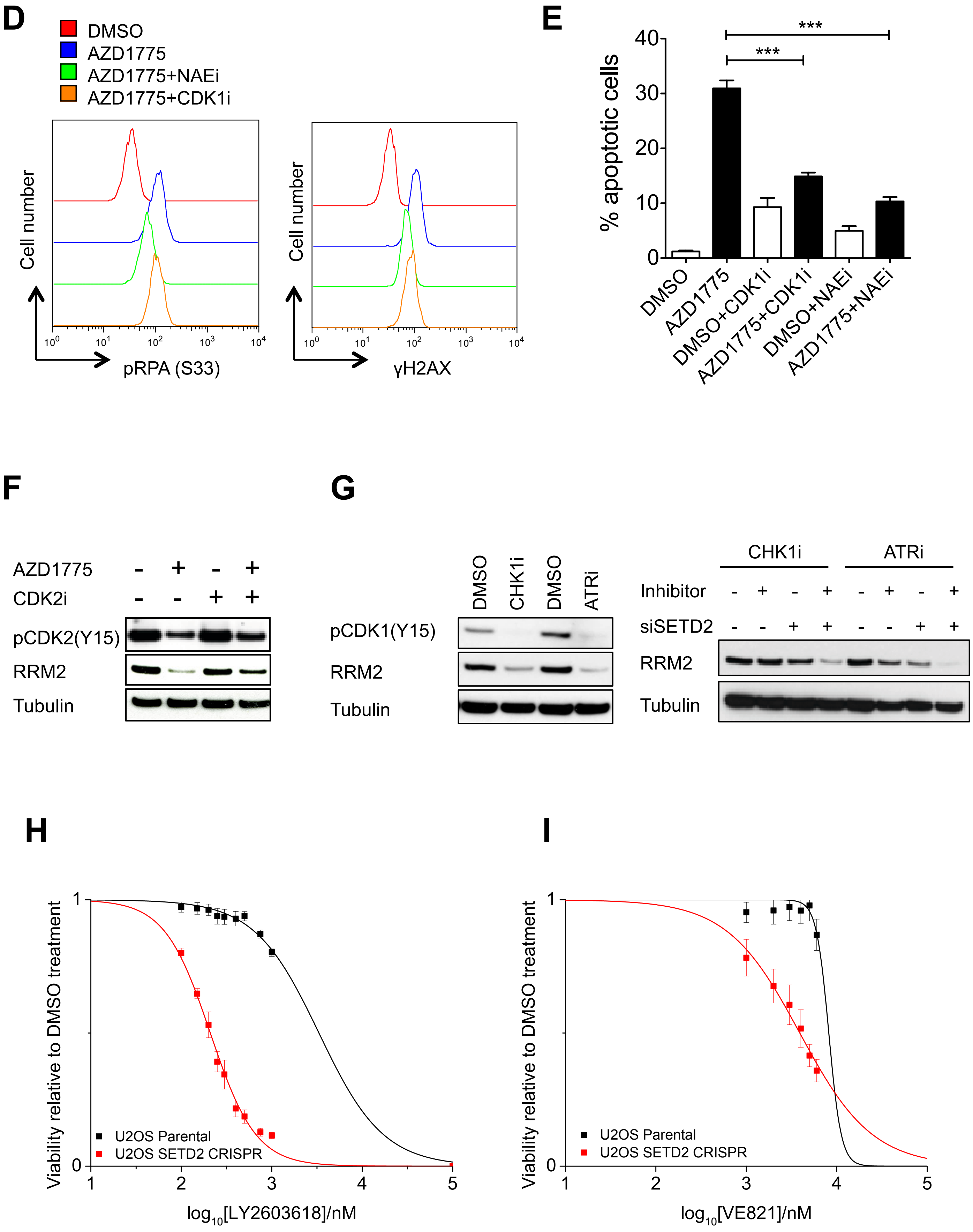
D
DMSO
AZD1775
AZD1775+NAEi
AZD1775+CDK1i
Cell number
pRPA (S33)
γH2AX
E
% apoptotic cells
***
***
DMSO
AZD1775
DMSO+CDK1i
AZD1775+CDK1i
DMSO+NAEi
AZD1775+NAEi
F
AZD1775
CDK2i
pCDK2(Y15)
RRM2
Tubulin
G
DMSO
CHK1i
DMSO
ATRi
pCDK1(Y15)
RRM2
Tubulin
CHK1i
ATRi
Inhibitor
siSETD2
RRM2
Tubulin
H
Viability relative to DMSO treatment
U2OS Parental
U2OS SETD2 CRISPR
log10[LY2603618]/nM
I
Viability relative to DMSO treatment
U2OS Parental
U2OS SETD2 CRISPR
log10[VE821]/nM

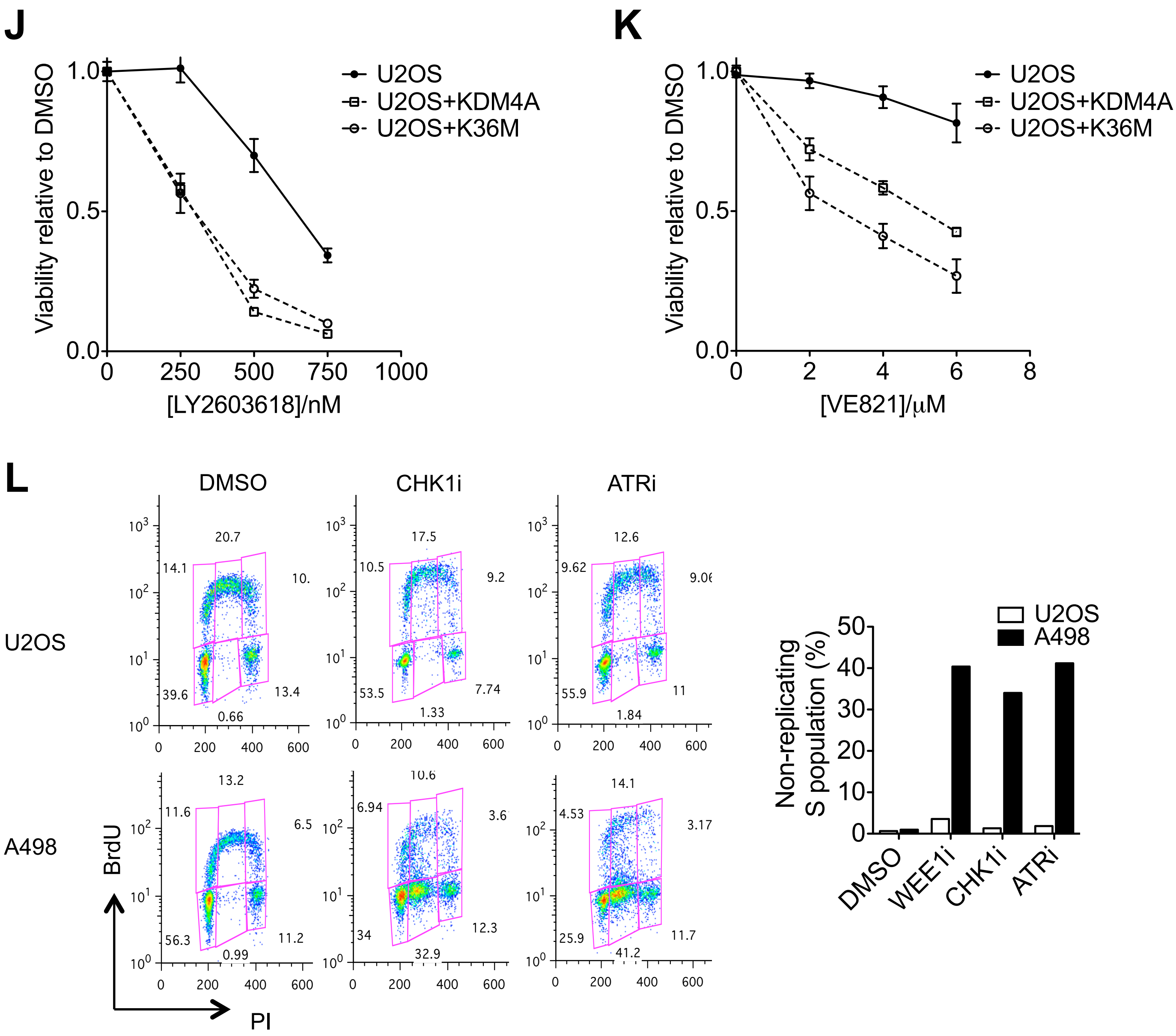
J
Viability relative to DMSO
1.0
0.5
0.0
0
250
500
750
1000
[LY2603618]/nM
U2OS
U2OS+KDM4A
U2OS+K36M
K
Viability relative to DMSO
1.0
0.5
0.0
0
2
4
6
8
[VE821]/μM
U2OS
U2OS+KDM4A
U2OS+K36M
L
DMSO
CHK1i
ATRi
U2OS
A498
BrdU
PI
Non-replicating S population (%)
U2OS
A498
DMSO
WEE1i
CHK1i
ATRi

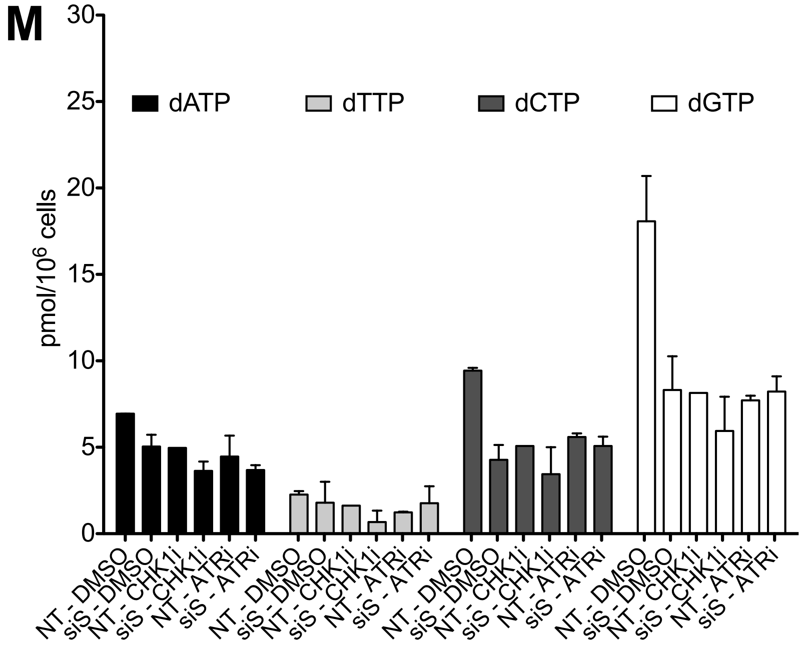
M
pmol/10^6 cells
dATP
dTTP
dCTP
dGTP
NT - DMSO
siS - DMSO
NT - CHK1i
siS - CHK1i
NT - ATRi
siS - ATRi

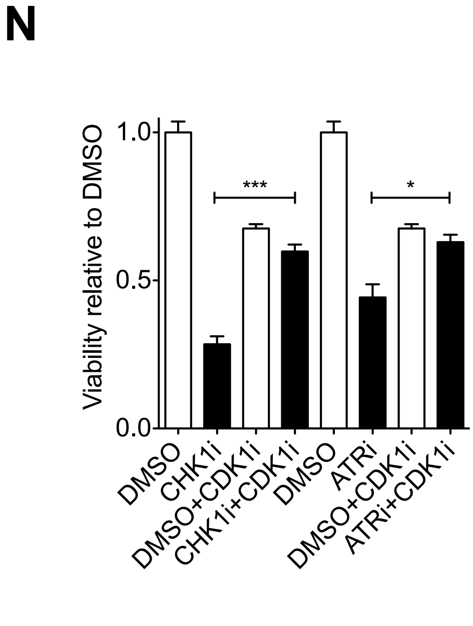
N
Viability relative to DMSO
1.0
0.5
0.0
***
*
DMSO
CHK1i
DMSO+CDK1i
CHK1i+CDK1i
DMSO
ATRi
DMSO+CDK1i
ATRi+CDK1i

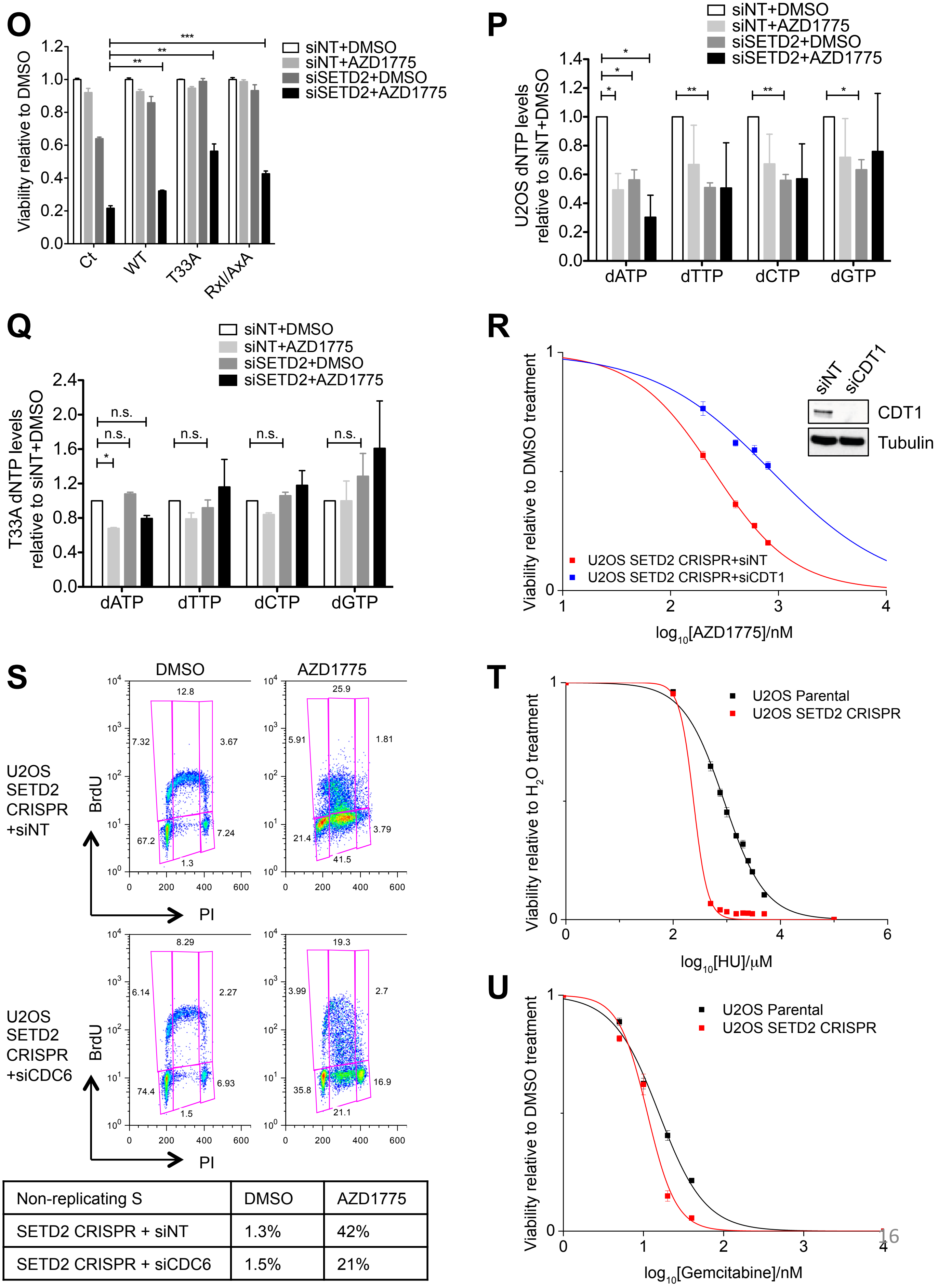

Non-replicating S	DMSO	AZD1775
SETD2 CRISPR + siNT	1.3%	42%
SETD2 CRISPR + siCDC6	1.5%	21%

Figure S4, related to Figure 4. WEE1 inhibition degrades RRM2 and increases aberrant origin firing.

(A) U2OS and U2OS CRISPR SETD2-knockout cells were treated with indicated concentrations of AZD1775 and harvested at indicated time. Lysis buffer contained protease inhibitor and phosphatase inhibitor. Western blot analysis of levels of phospho-CDK1 (Y15), phospho-RRM2 (T33) and RRM2. Tubulin serves as the loading control.
(B) Analysis of cell viability of U2OS CRISPR SETD2-knockout cells treated with DMSO or AZD1775 (200 nM) combined with either neddylation inhibitor MLN4924 (NAEi) (0.1 mmol/L), CDK1 inhibitor RO3306 (CDK1i) (10 μM) or CDK2 inhibitor CVT-313 (CDK2i) (3 μM). Cell viability was measured by Resazurin (72 hr).
(C) FACS analysis of BrdU incorporation in SETD2-deficient A498 cells treated with DMSO or AZD1775 (200 nM) combined with either neddylation inhibitor MLN4924 (NAEi) (0.1 mmol/L) or CDK1 inhibitor RO3306 (CDK1i) (10 μM) for 48 hr. Percentage of non-replicating S-phase cells are indicated.
(D) SETD2-deficient A498 cells were treated as in (C), FACS analysis of phospho-RPA (S33) and γH2AX levels.
(E) Percentage apoptosis of A498 cells after treatment described in (C).
(F) Western blots showing the levels of inhibitory CDK2-Y15 phosphorylation and RRM2 in A498 cells after treatment with AZD1775 (200 nM) and the CDK2 inhibitor CVT-313 (CDK2i) (3 μM) (24 hr).
(G) Cell were treated with DMSO, CHK1 inhibitor LY2603618 (500 nM) or ATR inhibitor VE821 (4 μM) for 24 hr. Western blots showing the levels of inhibitory CDK1-Y15 phosphorylation and RRM2 in A498 cells (left) and RRM2 levels in U2OS cells (right) transfected with control or SETD2 siRNA (siSETD2) 48 hr prior to inhibitor treatment.
(H) Viability of U2OS and U2OS CRISPR SETD2-knockout cells 5 days after CHK1 inhibitor LY2603618 treatment.
(I) Viability of U2OS and U2OS SETD2-knockout cells 5 days after ATR inhibitor VE821 treatment.
(J) Viability of U2OS cells stably expressing the dominant-negative H3.3K36M or overexpressing the demethylase KDM4A 5 days after treatment with CHK1 inhibitor LY2603618 (CHK1i).
(K) Viability of U2OS cells stably expressing the dominant-negative H3.3K36M or overexpressing KDM4A 5 days after treatment with ATR inhibitor VE821 (ATRi).
(L) FACS analysis of BrdU incorporation in U2OS and A498 cells following treatment with DMSO, CHK1 inhibitor LY2603618 (250 nM) or ATR inhibitor VE821 (2 μM) (48 hr). Quantification of the percentage of the non-replicating S-phase population is shown on the right.
(M) Measurement of dNTP levels in U2OS cells after transfection with either non-targeting siRNA (siNT) or SETD2 siRNA (siS) (72 hr) and treatment with either the CHK1 inhibitor LY2603618 (500 nM) or the ATR inhibitor VE821 (2.5 μM) for 24 hr. Absolute values of dNTP concentrations were shown.
(N) Viability of A498 cells 48 hr after treatment with either CHK1 inhibitor LY2603618 (500 nM) or ATR inhibitor VE821 (4 μM), with or without CDK1 inhibitor RO3306 (10 μM).
(O) U2OS cells stably expressing empty vector (Ct), wild-type RRM2 (WT), RRM2-T33A mutant (T33A) or RRM2-RxI/AxA mutant (RxI/AxA) were transfected with non-targeting siRNA (siNT) or SETD2 siRNA (siSETD2) 48 hr prior to DMSO or AZD1775 (200 nM) treatment for 4 days. Viability was measured by Resazurin.
(P) Measurement of dNTP levels in U2OS cells after transfection with either non-targeting siRNA (siNT) SETD2 siRNA (siSETD2) 48 hr and DMSO or AZD1775 (200 nM) treatment for 24 hr. Data are normalized to the control (siNT+DMSO).

(Q) Measurement of dNTP levels in U2OS cells stably expressing degradation-resistant RRM2 (T33A) after transfection with either non-targeting siRNA (siNT) or SETD2 siRNA (siSETD2) for 48 hr, and DMSO or AZD1775 (200 nM) treatment for 24 hr. Data are normalized to the control (siNT+DMSO).

(R) Viability curves of U2OS CRISPR SETD2-knockout cells transfected with either non-targeting siRNA (siNT) or CDT1 siRNA (siCDT1) (48 hr) and exposed to AZD1775 (5 days). Western blots showing the knockdown of CDT1 protein.

(S) FACS analysis of BrdU incorporation in U2OS CRISPR SETD2-knockout cells transfected with either non-targeting siRNA (siNT) or CDC6 siRNA (siCDC6) (48 hr) and exposed to DMSO or AZD1775 (48 hr). The percentage of non-replicating S-phase population are shown.

(T) Viability curves of U2OS and U2OS CRISPR SETD2-knockout cells after treatment with indicated concentrations of hydroxyurea (HU) (5 days).

(U) Viability curves of U2OS and U2OS CRISPR SETD2-knockout cells after treatment with indicated concentrations of gemcitabine (5 days).

All data in Figure S4 are presented as mean ± SEM, n = 3 independent experiments, p values are calculated by unpaired, two-tailed t-test or column statistics (one sample t-test) wherever appropriate. ***$p < 0.001$, **$p < 0.01$, *$p < 0.05$, n.s. not significant.

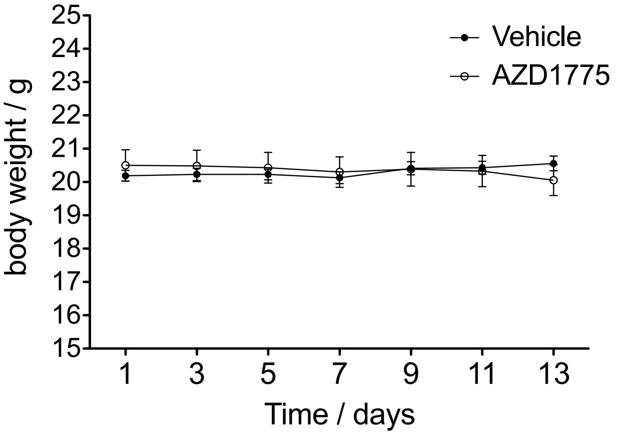

Figure S5, related to Figure 5. AZD1775 is well tolerated in the in vivo model.

Mean body weight of tumor-bearing mice treated with either vehicle or AZD1775 as described in Figure 5A. Data are presented as mean ± SEM, n = 7 mice.

Supplemental Experimental Procedures

Cell culture

U2OS (human osteosarcoma), A498 (human renal cell carcinoma) and RCC4 (human renal cell carcinoma) cell lines were cultured at 37 °C with 5% CO_2 in Dulbecco's modified Eagle's medium (DMEM) supplemented with 10% v/v fetal bovine serum (FBS) and penicillin (100 units/ml) / streptomycin (0.1 mg/ml). LB996 (human renal cell carcinoma) was obtained from Benoît Van den Eynde and was cultured in IMDM supplemented with 10% FBS, penicillin/streptomycin and G5 supplement (Invitrogen). All cell lines were routinely tested for mycoplasma and found to be negative.

Genomic validation of CRISPR SETD2 knockout cells

The CRISPR knockout cells were validated at the DNA level by genomic PCR and Sanger sequencing. Genomic DNA was extracted from U2OS parental and U2OS CRISPR SETD2-knockout cells. PCR was performed on the genomic DNA with primers located approximately 500 bp on each side of the target site. The PCR product was cleaned by Qiagen spin column and sequenced by Sanger sequencing. The sequences from U2OS parental, U2OS CRISPR, NCBI reference genome and target sequence were aligned using BioEdit ClustalW multiple sequence alignment tool. The amino acid translation was cross-checked with the amino acid sequence from the CCDS report.

Potential off-target sites of the gRNA was predicted using the MIT CRISPR design tool (http://crispr.mit.edu/). The top three predicted off-target sites were chosen and primers were designed to be approximately 500 bp on each side of the predicted sites. The primer sequences are listed below:

On-target Frd: TCCCATATTGGGAAGGATGA

On-target Rev: GGGGATAATTCCGATCCAGT

Predicted Off-target1 Frd: AGTGGACTTCGCTCCAGAAA

Predicted off-target1 Rev: AGAGTGGCAGGCTGTTCCTA

Predicted off-target2 Frd: TAGATGTTAGATGTGCCGGTGC

Predicted off-target2 Rev: GTTACCTCTGACTGTGAGCCCTT

Predicted off-target3 Frd: TTTTGAACCTGTCGCCAAGC

Predicted off-target3 Rev: ATCCGCCTGAATGTGGGTAG

Inhibitors

All inhibitors were dissolved in Dimethyl sulfoxide (DMSO) and stored at -80 ºC, and used according to the manufacturers instructions.

Inhibitor names	Target	Purchased from
AZD1775 (MK1775)	WEE1	Axon Medchem
LY2603618 (IC-83)	CHK1	Selleck Chemicals
VE821	ATR	Axon Medchem
KU55933	ATM	Tocris Bioscience

Resazurin assay for cell viability

Equal numbers of cells were seeded in a 96-well plate 24 hr before inhibitor treatment. DMSO (solvent for the inhibitors) was used as a negative control. The inhibitor was washed off after 3 days. 5 days after addition of inhibitors, the media were removed and fresh media containing Resazurin were added to each well. Resazurin is a nonfluorescent dye, which can be converted (by redox reaction) to a red fluorescent resorufin by living cells. The fluorescent signal is proportional to the number of living cells, and was measured by a fluorescence plate reader (BMG Labtech).

Assay for apoptosis

Equal numbers of cells were seeded in a 96-well plate 24 hr prior to inhibitor treatment. 48 hr after addition of inhibitors, Hoechst was added to the media. Hoechst stains the DNA, and the nuclei of apoptotic cells appear condensed and bright. Images were taken using Incell Analyzer (GE Healthcare) and the number apoptotic cells were counted using Incell Analysis software (GE Healthcare), and presented as a percentage of total number of cells.

Clonogenic survival assay

400 cells were plated per well in 6-well plates. 4 hr after plating, cells were treated with desired inhibitors at indicated concentrations. The inhibitors were washed off after 4 days and colonies were fixed and stained after 14 days. Plating efficiency (PE) and surviving fraction (SF) were calculated according to Nature Protocol.

Gene complementation

For expression of wild-type SETD2 in SETD2-deficient cells (A498), wild-type full length *SETD2* cDNA was purchased from Source Bioscience and inserted into pcDNA6.2-DEST mammalian expression plasmid (Invitrogen). After verification by sequencing, the plasmid was transfected into A498 cells by electroporation and an empty vector was used as control. Stable integrations were selected by Blasticidin. Generation of Doxycycline inducible KDM4A U2OS cells and stable H3.3K36M U2OS cells are described previously (Pfister et al., 2014), and are cultured in complete DMEM medium with appropriate selection.

RNAi

U2OS or A498 cells were transfected with siRNAs (10 nM final concentration) using RNAiMAX (Invitrogen) according to the manufacture's instructions. Medium was replaced 24 hr after transfection. The sequences of the siRNAs are listed below:

siSETD2#3 (si#3) (Dharmacon): GAAACCGUCUCCAGUCUGU

siSETD2#5 (si#5) (Dharmacon): UAAAGGAGGUAUAUCGAAU

siWEE1 (Dharmacon): AAUAGAACAUCUCGACUUA, AAUAUGAAGUCCCGGUAUA, GAUCAUAUGCUUAUACAGA, CGACAGACUCCUCAAGUGA

siCHK1 (Invitrogen): GGCUUGGCAACAGUAUUUCGGUAUA, CCCAGC-CCACAUGUCCUGAUCAUAU, UGCCAGUGAAGAUUGUAGAUAUGAA,

siCHK2 (Dharmacon): GUAAGAAAGUAGCCAUAAA, GCAUAGGACU-CAAGUGUCA, GUUGUGAACUCCGUGGUUU, CUCAGGAACUCUAUUCUAU

siATM (Invitrogen): GCGCAGUGUAGCUACUUCUUCUAUU

siATR (Invitrogen): GGGAAAUACUAGAACCUCAUCUAAA

siCDT1, siLEDGF, siCtIP, siCDT2, siCDC6, siBRCA2 are all purchased from Dharmacon ON-TARGET plus predesigned siRNAs.

shRNA transduction

Lentiviral particles containing shRNA for MUS81 was obtained from Dr Songmin Ying. 3 μl of the lentiviral particle was added per well of a 6-well plate. MUS81 knockdown was analyzed by Western blotting 72 hr after incubation.

Antibodies

PCNA (Santa Cruz, PC-10), RPA32 (Abcam, 2175), Histone H3 (Abcam, 10799), SETD2 (Abcam, 31358), H3K36me3 (Abcam, 9050) and KDM4A (Cell Signaling, 5328), RRM2 (Santa Cruz, sc-10844), RRM1 (Santa Cruz, sc11733), pRPA (S33) (Bethyl, A300-246A), γH2AX (Upstate, 05-636), Tubulin (Sigma, T5168).

Cell cycle analysis by BrdU incorporation

Cells were incubated in media containing 20 μM BrdU for 20 min at 37 °C and protected from light. Cells were collected by trypsinisation and fixed in ice-cold 70%

ethanol for at least 30 min. Since the anti-BrdU antibody recognizes single-stranded DNA, cells were incubated for 20 min at room temperature in 2M HCl (which denatures double-stranded DNA) containing 0.1 mg/ml pepsin (which digests nuclear proteins to expose DNA). Cells were washed once in PBS, followed by once in PBS containing 0.5% FBS and 0.5% Tween. Cells were incubated in the blocking buffer (PBS with 2% FBS) containing the anti-BrdU monoclonal antibody (BD Biosciences) (1:100 dilution) for 90 min at room temperature. Cells were washed twice with PBS and incubated in the blocking buffer containing the Alexa Fluor 488 secondary antibody (Invitorgen) (1:200 dilution) for 60 min at room temperature in the dark. Cells were washed in PBS and resuspended in PBS containing 0.1 mg/ml propidium iodide and analysed by FACS Scan (Becton Dickinson). S-phase length was calculated according to established protocol (Begg et al., 1985).

DNA fiber assay using CldU/IdU labelling

Cells were labelled for 20 min in medium containing 25 μM CldU followed by 20 min in medium containing 250 μM IdU at 37 °C. Cell were collected by trypsinization and resuspended in ice-cold PBS at the concentration of 5×10^5 cells/ml. 2 μl of the suspension was pipetted onto a microscope slide, let dry before adding 7 μl of the spreading buffer (200 mM Tri-HCl pH7.4, 50 mM EDTA, 0.5% SDS) and mixed. The DNA was allowed to run down the slide slowly, air-dried and fixed in methanol/acetic acid (3:1) for 10 min. The slides were washed with H_2O and denatured in 2.5M HCl for 1 hr 15 min. The slides were washed with PBS and incubated in blocking solution (PBS containing 1% BSA and 0.1% Tween) for 1 hr, and incubated in Rat anti-BrdU antibody (1:1000 dilution) and Mouse anti-BrdU antibody (1:1000 dilution) overnight at 4°C. The slides were washed with PBS and fixed in 4% paraformaldehyde for 10 min. After rinsing, the slides were incubated in anti-Rat AlexaFluor 555 antibody (1:500 dilution) and anti-Mouse AlexaFluor 488 antibody (1:500 dilution) for 1.5 hr at room temperature. After washing, the slides

were mounted in Vectashield and analysed using the Zeiss LSM780 confocal microscope and ImageJ.

Quantitative RT-PCR

Total RNA (1 μg) was purified using the RNeasy kit (Qiagen) and cDNA was prepared using the SuperScript RT–PCR system (Invitrogen). Quantitative RT-PCR was performed using Absolute Blue QPCR SYBR low ROX Mix (Thermo Scientific) according to the manufacturer's protocol. Reactions were carried out in duplicate for each target transcript using a 7500 Fast Real-Time PCR System (Applied Biosystems). The comparative CT method was applied for quantification of gene expression, and values were normalized against GAPDH as control. Results were expressed as fold change in mRNA levels. The following primers were used:

RRM2 for (5'-TTTAAAGGCTGCTGGAGTGAGG-3'),

RRM2 rev (5'-GCAGCTGCTTTAGTTTTCGGCT-3'),

SETD2 for (5'-CTTTCTGTCCCACCCCTGTC-3'),

SETD2 rev (5'-CCTTGACCTCGATGGCTT-3'),

BRCA2 for (5'- GCGCGGTTTTTGTCAGCTTA-3'),

BRCA2 rev (5'- TGGTCCTAAATCTGCTTTGTTGC-3'),

GAPDH for (5'-AGCCACATCGCTCAGACAC-3'),

GAPDH rev (5'-GCCCAATACGACCAAATCC-3').

ChIP-qPCR analysis

Chromatin immunoprecipitations were performed as described previously (Zalmas et al., 2013). Species-matched non-specific immunoglobulins (NS) or antibodies against H3K36me3 (Abcam, ab 9050), RNA Polymerase II (Abcam, ab 817), phoshpho RNA Pol II ser5 (Abcam, ab 5131), RNA Polymerase II ser2 (Abcam, ab 5095) and TAF6 (Abcam, ab 76922) were used for ChIP. DNA was amplified with

Paq5000 polymerase (Agilent) with primers pairs listed below. ChIP-qPCR data are presented as percentage of input. ΔCt= CtIP - corrected Ct(input), where corrected Ct(input)=Ct(input) - log2(10) to account for the 1/10 dilution factor of the input; IP = immunoprecipitation, input = chromatin. The qTR-PCR primers are listed below:

RRM2 promoter for: GGCAAATCAGAAAGCCACATAG

RRM2 promoter rev: GTACTACTCATTGGGCGTCAA

RRM2 promoter primer 2 for: CTCAGCGGCCCTAACTTT

RRM2 promoter primer 2 rev: CTTTCGATCCGTGTCCCT

RRM2 exon 3 for: GCCGCTTTGTCATCTTCC

RRM2 exon3 rev: AGGAAGCCTCTGCCTTCTTA

RRM2 Intron 4 for: TTGACGTTGACGATCTGAGG

RRM2 Intron 4 rev: ACACACAGATGCACTCAGCA

RRM2 Intron 5, for: AGGTGGGCACCAGAATAAAG

RRM2 Intron 5, rev: GGTCAGGAAAGCAAATCCAT

RRM2 Intron 7, for: AAAGCCAGGAGCATGAACTC

RRM2 Intron 7, rev: TCCCAATCCAGTAAGGAAGG

Pharmacodynamics studies

For pharmacodynamics studies, sections (4 μm) from paraformaldehyde-fixed paraffin embedded tumors were deparaffinized and rehydrated. Staining for cleaved caspase-3 (antibody #9661, 1:600 dilution, Cell Signaling Technology) and gH2AX (antibody #05-636, 1:2000, Millipore) was carried out using the DAKO Real™ Envision Detection System (Dako, Cambridge, UK) with diaminobenzidine (DAB) as a chromogen.

Supplemental References

Bamford, S., Dawson, E., Forbes, S., Clements, J., Pettett, R., Dogan, A., Flanagan, A., Teague, J., Futreal, P. A., Stratton, M. R., and Wooster, R. (2004). The COSMIC (Catalogue of Somatic Mutations in Cancer) database and website. Br J Cancer *91*, 355-358.

Zalmas, L. P., Coutts, A. S., Helleday, T., and La Thangue, N. B. (2013). E2F-7 couples DNA damage-dependent transcription with the DNA repair process. Cell Cycle *12*, 3037-3051.
